# OSH45, a homeobox transcription factor, coordinates low-phosphate adaptation in rice

**DOI:** 10.3389/fpls.2025.1654599

**Published:** 2025-08-28

**Authors:** Hong Lu, Kangming Jin, Yuelin Luo, Yunrong Wu, Yu Liu, Jiming Xu, Chuanzao Mao

**Affiliations:** ^1^ State Key Laboratory of Plant Environmental Resilience, College of Life Sciences, Zhejiang University, Hangzhou, China; ^2^ Hainan Institute of Zhejiang University, Yazhou Bay Science and Technology City, Sanya, Hainan, China

**Keywords:** *Oryza sativa* L., phosphate starvation, knotted1-like homeobox protein, transcriptome, phosphate transporter

## Abstract

Phosphorus (P), an essential macronutrient critical for plant growth and development, faces significant availability constraints in agricultural soils, substantially limiting crop yield potential. Transcription factors (TFs) play pivotal roles in phosphate (Pi) starvation responses in plants. In this study, we identified *OSH45* (*Oryza sativa homeobox 45*), a homeobox domain TF in rice (*Oryza sativa* L.), which was strongly induced in roots under Pi starvation. Subcellular localization assays indicated that OSH45 is a nuclear localized protein. *OSH45* overexpression transgenic plants exhibited enhanced low-Pi tolerance, characterized by significantly higher Pi concentrations and increased shoot and root biomass compared to wild type (WT) under Pi-limited conditions. Whereas *osh45* loss-of-function mutants displayed no significant difference in shoot and root biomass compared to WT under both Pi-sufficient and Pi-limited conditions, but showed lower Pi concentration under Pi-sufficient conditions. Through transcriptomic profiling, 2,406 differential expressed genes (DEGs) were identified in *OSH45* overexpression plants versus WT under Pi-sufficient conditions. About 38% of Pi starvation-induced (PSI) genes were upregulated and 25% of Pi starvation-suppressed (PSS) genes were downregulated in *OSH45* overexpression plants. The expression of phosphate transporters, such as *OsPT1*, *OsPT2*, *OsPT4*, *OsPT8*, and acid phosphatases was upregulated, while the expression of Pi signaling repressors *OsSPX1-3* was suppressed in *OSH45* overexpression plants. Conversely, *osh45* displayed decreased expression of *OsPT1* and *OsPT8* compared to WT. Altogether, our findings demonstrated that OSH45 is a novel TF involved in Pi deficiency response, regulating a set of Pi starvation responsive (PSR) genes to optimize plant adaptation to Pi-limited environments. This mechanism provides a strategic target for engineering Pi-efficient crops.

## 1 Introduction

Phosphorus (P) is an essential macronutrient for plant growth and development, which is primarily absorbed as inorganic phosphate (Pi) in the forms H_2_PO₄^−^, HPO₄²^−^, or PO₄³^−^ depending on soil pH (Brinch-Pedersen et al., 2002). Pi scarcity severely limits agricultural productivity, affecting approximately 50% of global arable lands (Lynch, 2011; Liu et al., 2024). Phosphorus fertilization is a common strategy for improving crop yields in Pi-limited soils. However, due to its low diffusion coefficient and strong chelation with metal ions, less than 25% of the phosphate fertilizer is directly available to crops (Johnston et al., 2014; Puga et al., 2024). Elucidating the molecular mechanisms governing Pi signaling pathways is crucial for engineering crops with improved phosphorus-use efficiency (PUE), which could significantly reduce fertilizer dependency while ensuring sustainable agricultural production (Paz-Ares et al., 2022; Madison et al., 2023).

To cope with Pi limitation, plants have evolved multifaceted strategies to enhance Pi acquisition and utilization efficiency (Ham et al., 2018; Wang et al., 2021; Poirier et al., 2022). Under Pi-deficient conditions, plants remodel root architecture to increase Pi foraging capacity and fine-tune the activity of phosphate transporters (PTs) through transcriptional and post-transcriptional regulation, thereby optimizing Pi uptake and redistribution (Wang et al., 2021). Although significant advances have been made to elucidate the molecular components of Pi deficiency responses, the detailed mechanism underlying plant adaptation to Pi limitation remains unclear. Notably, transcription factors (TFs) act as central players in orchestrating plant adaptation to Pi scarcity. In *Arabidopsis*, the Myeloblastosis (MYB)-type regulators PHR1 (Phosphate Starvation Response 1) and its homologs PHL1-4 serve as master regulators of Pi starvation signaling, while their orthologs OsPHR1-4 function as master regulators of Pi starvation response (PSR) in rice. These MYB-CC type TFs activate subsets of Pi starvation induced (PSI) genes by binding to the P1BS motif (GNATATNC) in target gene promoters (Rubio et al., 2001; Bustos et al., 2010; Chiou and Lin, 2011; Guo et al., 2015; Sun et al., 2016). The PHR-regulated genes coordinate Pi uptake, assimilation, and reallocation, while PHR activities are post-translationally modulated by SPX (SYG1/Pho81/XPR1) proteins that act as Pi sensors (Wang et al., 2014; Lv et al., 2014; Zhou et al., 2021; Guan et al., 2022). Alongside core PHR regulators, additional MYB family members participate in the regulation of Pi homeostasis. In rice, OsRLI1 (Regulator of leaf inclination 1) is involved in regulating leaf angle based on Pi status and activating PSR genes under Pi deficiency (Ruan et al., 2018; Zhang et al., 2021). OsMYB2P-1, OsMYB4P, and OsMYB5P regulate Pi homeostasis, partly through transcriptional control of phosphate transporter genes (Dai et al., 2012; Yang et al., 2014; Wu et al., 2018). Additional TF families including WRKY, GRAP, and bHLH are also involved in Pi signaling regulation. In *Arabidopsis*, AtWRKY75 and AtWRKY45 activate *AtPHT1* genes through W-box binding, while in rice, OsWRKY21, OsWRKY74 and OsWRKY108 activate *OsPT* genes via the same mechanism (Devaiah et al., 2007; Zhang et al., 2020; Dai et al., 2016). Conversely, OsWRKY10 represses *OsPT2* under Pi-sufficient conditions (Wang et al., 2022). GARP family TF NIGT1 (Nitrate-inducible GARP-type transcriptional repressor 1) coordinates nitrogen (N)-P crosstalk, enhancing Pi absorption while suppressing nitrate uptake (Wang et al., 2023a). bHLH TF OsPTF1 confers low-Pi tolerance (Yi et al., 2005), while OsbHLH6 interacts with OsSPX4 to release OsPHR2, thereby activating PSI genes (He et al., 2021). This multilayered transcriptional network underscores the complexity of Pi signaling regulation. Characterization of novel TFs will advance our understanding of the plant Pi signaling network and provide strategies for improving PUE.

Homeodomain (HD) TFs are pivotal regulators governing plant development, which are phylogenetically categorized into homeodomain-leucine zipper (HD-Zip) and three-amino-acid loop extension (TALE) families. The TALE superfamily includes knotted1-like homeobox (KNOX) transcription factors critical for meristem maintenance, organogenesis, and phytohormone regulation (Hake et al., 2004). The KNOX subfamily were divided into class I (Shoot Apical Meristem (SAM)-specific, essential for meristem maintenance) and class II (broader expression, functional diversification) based on phylogeny and expression (Furumizu et al., 2015). Class I KNOX genes, such as *STM* (*Shoot meristemless*) in *Arabidopsis*, *KN1* (*Knotted 1*) in maize, *OSH1* (*Oryza sativa* homeobox 1) in rice, have been well characterized for their roles in SAM development (Tsuda et al., 2011; Chen et al., 2023; Tsuda et al., 2014), whereas the functions of class II KNOX proteins remain less explored. In *Arabidopsis*, class II members (AtKNAT3, AtKNAT4, AtKNAT5 and AtKNAT7) participate in cytokinin response and secondary cell wall biosynthesis (Wang et al., 2020b). In rice, the class II subfamily comprises HOS58 (Homeobox *oryza sativa* 58), HOS59, OSH45, and OsKNAT7/HOS66. Current evidence shows OsKNAT7 regulates cell wall biosynthesis and grain size through interactions with OsNAC31 and OsGRF4 (Wang et al., 2020b; Yu, 2019). Recently, ChIP-seq analyses identified HOS59 as a transcriptional repressor of grain size regulators *OsSPL13* and *OsSPL18*, yet comprehensive mechanistic insights into KNOX II TFs remain scarce (Sheng et al., 2022). Although several HD domain proteins were known as developmental regulators, whether HD domain proteins are involved in plant adaptation to P nutrient limitation remains unexplored.

Here, we identify *OSH45*, a class II KNOX TF transcriptionally upregulated under P deficiency. We create *CRISPR/Cas9* mutants and overexpression transgenic lines in rice, and demonstrate that OSH45 promotes Pi acquisition by modulating the expression of genes encoding phosphate transporters and phosphatases, while suppressing the expression of Pi signaling repressors *OsSPXs*. These findings establish OSH45 as a new key regulator of PSR to optimize plant adaptation to Pi-limited environments, which will benefit engineering Pi-efficient crops.

## 2 Materials and methods

### 2.1 Plant materials and growth conditions

The wild-type (WT) *japonica* rice (*Oryza sativa* L.) cultivar ‘Nipponbare’ (NIP) was used in this study. All transgenic lines were generated in the NIP background. Rice plants were hydroponically grown in a glasshouse under controlled conditions: 30/22°C (day/night), 60-70% relative humidity, and a 14 h light/10 h dark photoperiod with a light intensity of ~300 μmol m^-2^ s^-1^ provided by spectrum-tunable LED plant-growth lamps. Hydroponic cultivation was performed using full-strength Kimura nutrient solution as previously described (Wang et al., 2020a). Pi treatments were set as: Pi-sufficient (HP, 200 μM Pi), Pi-limited (LP, 10 μM Pi), and Pi-deficient (–P, 0 μM Pi). Solutions were prepared by replacing the appropriate amount of KH_2_PO_4_ with an equimolar concentration of KCl. The pH of the nutrient solution was adjusted to 5.5 using 1 M HCl or 1 M NaOH.

### 2.2 Phylogenetic analysis of the KNOX subfamily

To identify close homologs of OSH45 in the rice genome, the full-length OSH45 protein sequence was queried against the NCBI database using BLASTP (https://www.ncbi.nlm.nih.gov/). Candidate homologs with ≥ 80% query coverage and ≥ 50% identity were selected for further analysis. Multiple sequence alignment of the retrieved full-length protein sequences was performed with Clustal X.

### 2.3 Vector construction and generation of transgenic plants

To generate the *OSH45* overexpression vector, the 834-bp coding sequence (excluding the stop codon) of *OSH45* (*Os08g0292900*) was amplified from NIP cDNA using KOD-FX DNA polymerase (Toyobo, Japan). The amplified fragment was subsequently cloned into a modified *pCAMBIA1300-sGFP* vector under the control of the cauliflower mosaic virus *35S* promoter (Lv et al., 2014), resulting in a C-terminal GFP fusion protein. The two overexpression lines obtained after backcrossing with NIP were designated *OSH45*-*OE1* and *OSH45*-*OE2*. For *osh45* knockout mutants, a CRISPR-Cas9 system was employed following established protocols (Ma et al., 2015). Two target specific single-guide RNAs (sgRNAs; sequences listed in **Supplementary Data Sheet 1**) were designed and ligated into the *pYLCRISPR/gRNA* vector. The sgRNA cassettes were then ligated into the *pYLCRISPR/Cas9-MN* binary vectors as previously described (Ma et al., 2015). All constructs were validated by Sanger sequencing prior to transformation. Transgenic rice plants were generated via *Agrobacterium tumefaciens* (strain *EHA105*)-mediated transformation of embryogenic calli derived from mature NIP seeds, following standard procedures (Chen et al., 2015). The primers used for vector construction are provided in **Supplementary Data Sheet 1**.

### 2.4 Subcellular localization

For subcellular localization analysis, the *35S::OSH45-GFP* construct was transiently expressed in *Nicotiana benthamiana* leaves via *Agrobacterium tumefaciens* -mediated infiltration (strain *EHA105*). Fluorescence signals from root tips of 10-day-old transgenic rice seedlings harboring the *35S::OSH45-GFP* transgene were observed using a confocal laser-scanning microscope (LSM710, Zeiss). GFP signals were excited with a 488-nm argon-ion laser, and emission was collected between 493 to 542 nm. For DAPI, signals were excited with a 405-nm laser, and emission was collected between 420 to 480 nm. A 25 × water-immersion objective was used for confocal imaging.

### 2.5 RNA-sequencing and gene expression analysis

Seven-day-old NIP, *osh45* and *OSH45* overexpression seedlings cultured in full-strength Kimura nutrient solution were transferred to –P (0 μM Pi) and HP (200 μM Pi) solutions and cultured for a further 7 d. Roots from six independent plants were harvested, pooled together as one biological replicate, and used for RNA-sequencing (RNA-seq). Three biological repeats were performed per line and treatment. RNA-seq experiments were performed by LC-Bio Technologies Co., Ltd (Hangzhou, China). Total RNA was extracted using TRIzol reagent (Thermofisher, 15596018). mRNA libraries were constructed following the Illumina TruSeq Standed mRNA protocol and sequenced on Novaseq™ 6000 instrument. All paired-end reads were mapped to the *Oryza sativa* cv. Nipponbare reference genome using HISAT2 (v2-2.2.1). The rice reference genome and gene model annotations files (GFF files) were obtained from The Rice Annotation Project database (https://rapdb.dna.affrc.go.jp/). The raw sequencing data were deposited in the Genome Sequence Archive in National Genomics Data Center, China National Center for Bioinformation/Beijing Institute of Genomics, Chinese Academy of Sciences (Accession No. CRA026415) (https://ngdc.cncb.ac.cn/gsa).

The mapped reads of each sample were assembled using StringTie (http://ccb.jhu.edu/software/stringtie/) with default parameters. After the final transcriptome was generated, StringTie and ballgown were used to estimate the expression levels of all transcripts and perform expression abundance for mRNAs by calculating FPKM (fragment per kilobase of transcript per million mapped reads) value. The R package DESeq2 was used for analyzing differentially expressed genes (DEGs). The genes with the parameter of q-value below 0.05 and absolute fold change ≥ 2 were considered as DEGs. DEGs were then subjected to enrichment analysis of Gene Ontology (GO) functions and KEGG pathways. Clustered genes were assigned to biological process categories based on GO analysis using the GO database (http://geneontology.org/). GO terms with q-value < 0.05 were considered significantly enriched for DEGs. Heatmaps and Venn diagrams were visualized using TBtools (Chen et al., 2020).

### 2.6 RT-qPCR analysis

Total RNA was isolated from roots of related plants using TRIzol reagent (Thermo Fisher Scientific) according to the manufacturer’s instructions. First-strand cDNA synthesis was initiated from 2 μg of total RNA with a PrimeScript™ RT reagent kit with gDNA Eraser (RR047A; Takara, Japan). qPCR was performed using SYBR Green I Master (Roche) on a LightCycler 480 Real-Time PCR system according to the manufacturer’s instructions. *OsACTIN1* (*Os03g0718100*) was used to normalize the relative expression levels. Primers used for qPCR assays are listed in **Supplementary Data Sheet 1**.

### 2.7 Measurement of the concentration of Pi, total P, and other nutrient elements

Cellular Pi concentrations were measured as described previously (Wang et al., 2023b). Briefly, leaves, shoots and roots of 28-day-old seedlings hydroponically cultivated in Kimura nutrient solution containing 200 μM Pi were harvested. Pi concentrations were determined using a continuous flow analyzer (SAN++, SKALAR, Breda, the Netherlands) with molybdenum blue detection at 880 nm.

All plants, including WT and *OSH45* mutants and overexpression lines (BC_1_F_2_ transgenic lines), were harvested after 4 weeks’ growth in Kimura nutrient solution with 200 μM Pi, separated into roots and shoots, rinsed thoroughly with deionized water, and oven dried at 70°C for 72 h to constant mass. Total elemental analysis was conducted on dried tissues. Samples underwent microwave-assisted digestion (Mars 6, CEM Corporation) with concentrated 68% HNO_3_ (v/v) and 30% H_2_O_2_ (v/v) in a 5:1 (v/v) ratio. Phosphorus (P), potassium (K), iron (Fe), calcium (Ca), and copper (Cu) concentrations were determined by inductively coupled plasma-optical emission spectrometry (Optima 7300DV; Perkin-Elmer).

### 2.8 Statistical analyses

Statistical analyses were conducted using the program SPSS Statistics v22.0 (IBM). Data visualization was generated using GraphPad Prism 8.0 (GraphPad Software).

## 3 Results

### 3.1 OSH45 is a Pi starvation-responsive TF

**Figure 1:**
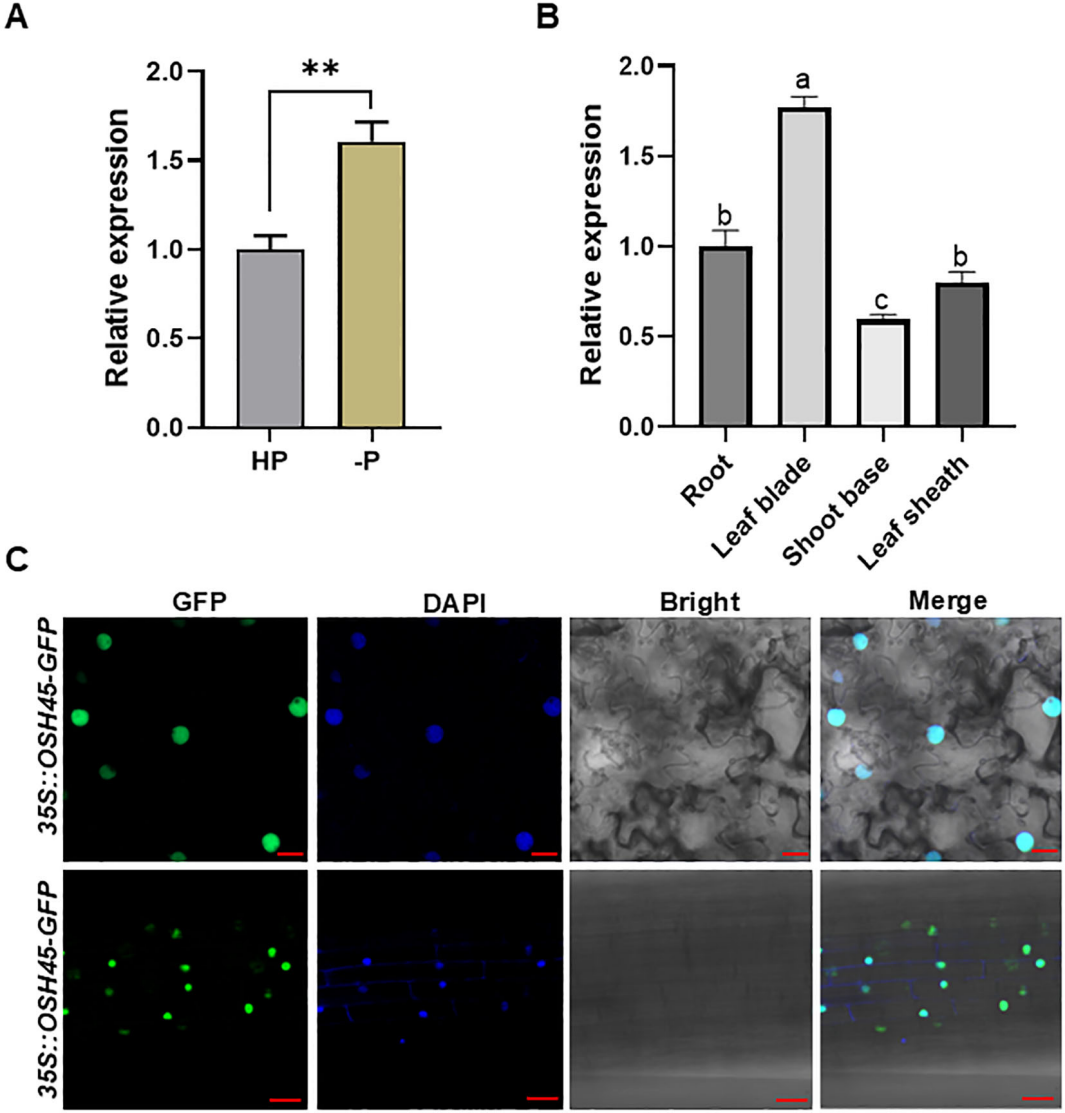
Expression pattern of *OSH45* and subcellular localization of OSH45. **(A)** Expression of *OSH45* in wild type (NIP) under HP (200 μM) or -P (0 μM Pi) treatment for 7 d. Seedlings were grown under normal hydroponic conditions for 7 d before -P treatment. **(B)** The relative expression of *OSH45* in different tissues of 7-day-old rice seedlings under Pi-sufficient conditions revealed by RT-qPCR. *ACTIN1* was used as an endogenous control in **(A, B)**. Data were normalized to the expression of *OSH45* in the NIP under Pi-sufficient conditions (which was set to 1). Data are means ± SD (*n*=3); asterisks in **(A)** indicate significant difference (**P < 0.01; Student’s *t*-test). Different lowercase letters in **(B)** indicate significant differences (*P* < 0.05; one-way ANOVA). **(C)** GFP fluorescence in *N.benthamiana* leaf cells transformed with *35S::OSH45-GFP* construct (upper panel), and in root epidermal cells of 10-d-old transgenic rice seedlings harboring *35S::OSH45-GFP* (lower panel). The blue signals indicate cell nucleus that were specifically stained with DAPI. Scale bars, 20 μm.

**Figure 2:**
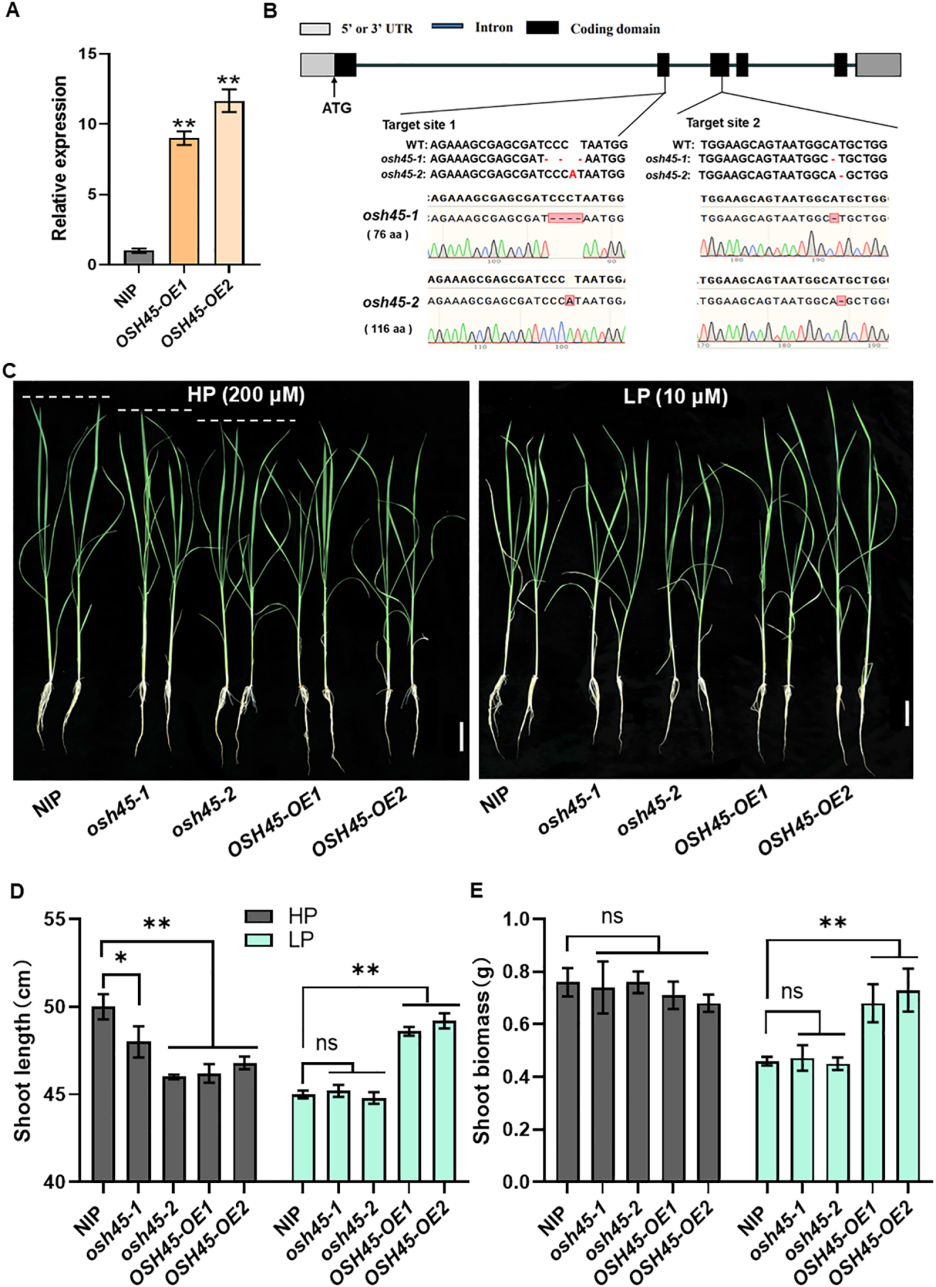
Phenotypes of *OSH45* mutants and overexpression transgenic lines under different Pi conditions. **(A)** Expression of *OSH45* in two independent *OSH45* overexpression transgenic lines. Seedlings were grown under nutrient-replete conditions for 10 d. *ACTIN1* was used as an endogenous control, *OSH45* expression in the NIP was set to 1. Date are means ± SD (*n*=3). **(B)** Target sites and mutated sequences in *osh45-1* and *osh45-2* mutants generated using CRISPR/Cas9. **(C)** Phenotypes of 21-day-old wild-type (NIP), *osh45* mutants, and *OSH45* overexpression lines. Scale bars, 5 cm. **(D, E)** Shoot length **(D)** and shoot biomass **(E)** of related plants. Seven-day-old plants cultured in Pi-sufficient solution were transferred to HP (200 μM) or LP (10 μM) conditions and grown for another 14 d. Date are means ± SD (*n*=10); asterisks indicate significant differences compared to NIP (ns, no significant difference, *P < 0.05, **P < 0.01; Student’s *t*-test).

To discover novel regulators in plant adaptation to Pi limitation, we identified *OSH45*, which is significantly upregulated under Pi deficiency, from the Plant Public RNA-seq Database (**Supplementary Figure S1**). OSH45, which contains a homeodomain (HD) together with characteristic KNOX1 and KNOX2 subdomains, is classified within the KNOX subfamily of HD transcription factors (**Supplementary Figure S2**). Sequence alignment of OSH45 homologs in rice (retrieved via BLASTP with ≥ 50% sequence identity and ≥ 80% query coverage) demonstrated that the OSH45 homologs contain conserved C-terminal HD domains but divergence in N-terminal sequences (**Supplementary Figure S2**). To validate whether *OSH45* responds to Pi deficiency, we performed RT-qPCR analysis using RNA extracted from roots of NIP under Pi-sufficient or Pi-deficient conditions. The result showed that the transcript level of *OSH45* was significantly upregulated under Pi deficiency (**Figure 1A**), consistent with the result from RNA-seq database. RT-qPCR analysis of *OSH45* expression across various tissues revealed that *OSH45* is ubiquitously expressed in different tissues, with a significantly higher expression level in the leaf blade than in the root, shoot base, and leaf sheath (**Figure 1B**). To determine the subcellular localization of OSH45, the *OSH45-GFP* fusion construct was transiently expressed in *N. benthamiana* leaves via *Agrobacterium*-mediated transformation. The GFP fluorescence was predominantly accumulated in the nucleus (**Figure 1C**), indicating that OSH45 is a nuclear-localized protein. Furthermore, two independent *OSH45* overexpression transgenic lines (*OSH45-OE1/OE2*), which exhibited significantly elevated *OSH45* transcript levels (**Figure 2A**), displayed predominantly nuclear localized GFP fluorescence (**Figure 1C**), consistent with OSH45’s predicted function as a transcription regulator (Tamaoki et al., 1995).

### 3.2 *OSH45* promotes plant growth under Pi starvation

To investigate the role of OSH45 in rice growth under Pi-limited conditions, we generated loss-of-function mutants in the NIP background using clustered regularly interspaced short palindromic repeat (CRISPR)/Cas9-mediated genome editing with two single-guide RNAs (sgRNAs) targeting the exon regions of *OSH45*. Two independent homozygous mutant lines, designated *osh45-1* and *osh45-2*, were identified and used for further analyses. *osh45-1* exhibited a 4-bp deletion in the first sgRNA target site and a 1-bp deletion in the second target site, while *osh45-2* exhibited a 1-bp insertion and a 1-bp deletion in the first and second target sites, respectively (**Figure 2B**). The mutants were backcrossed with NIP, and the homozygous *osh45-1* or *osh45-2* lines were identified from the BC1F2 to rule out the potential off-target mutations. To investigate the role of OSH45 in rice adaptation to Pi availability, 7-day-old NIP, *osh45*, and *OSH45* overexpression lines were treated hydroponically under Pi-sufficient (HP; 200 μM) or Pi-limited (LP; 10 μM) conditions for 14 d. Under HP conditions, no significant differences in root length or shoot biomass were observed among WT, mutant, and overexpression lines (**Figures 2C–E**; **Supplementary Figure S3**). However, both mutant and overexpression lines exhibited a significant reduction in plant height compared to WT (**Figures 2C, D**). Under LP conditions, the *OSH45* overexpression lines displayed superior growth performance compared to the WT, with an 8-10% increase in plant height and 47-58% increase in shoot biomass (**Figures 2C–E**). Notably, the *osh45* plants showed no significant difference in shoot length, shoot biomass, root length and root biomass compared to the WT under LP conditions (**Figures 2C–E**; **Supplementary Figure S3**). These results suggested that *OSH45* overexpression lines are tolerant to LP conditions compared to WT. After 21 d LP treatments, the *OSH45* overexpression lines showed significantly higher shoot and root fresh weight compared to WT. Conversely, the *OSH45* overexpression lines exhibited lower shoot and root fresh weight compared to WT under HP conditions (**Supplementary Figure S4**). Collectively, these results demonstrate that *OSH45* overexpression enhances rice growth under Pi-limited conditions.

### 3.3 OSH45 regulates Pi acquisition in rice

**Figure 3:**
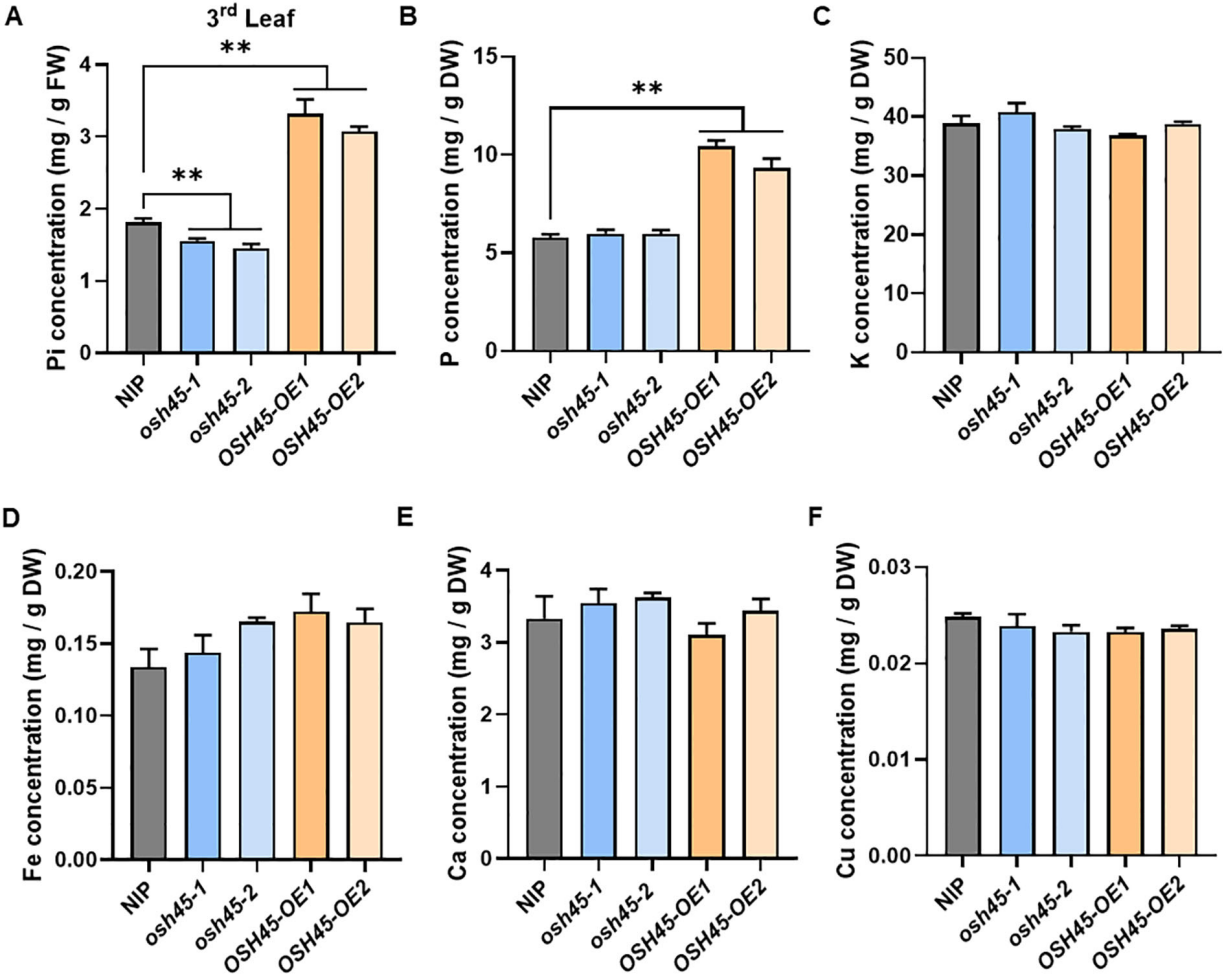
Pi concentration and element contents in *osh45* mutants and *OSH45* overexpression plants. **(A)** Leaf Pi concentration; **(B–F)** Total P concentration **(B)**, K concentration **(C)**, Fe concentration **(D)**, Ca concentration **(E)**, and Cu concentration **(F)** in the shoot of plants cultured in nutrient-sufficient conditions for 28 d. Date are means ± SD (*n*=5); asterisks indicate significant differences compared to wild-type NIP (**P < 0.01; Student’s *t*-test).

To investigate the potential role of OSH45 in Pi acquisition, Pi concentrations in the leaves of 28-day-old WT, *osh45*, and *OSH45* overexpression lines grown under HP conditions were quantified. The *osh45* showed a slight reduction in leaf Pi concentrations compared to the WT, whereas *OSH45* overexpression lines displayed 80-90% increase in Pi concentrations compared to the WT (**Figure 3A**). Whole-plant Pi concentration analysis further revealed that *OSH45* overexpression plants increased shoot Pi concentration by approximately 30% and significantly enhanced root Pi accumulation compared to WT. Although the root Pi concentration did not differ significantly between *osh45* and the WT, the shoot Pi concentration was reduced in *osh45* plants (**Supplementary Figure S5**). Similarly, *OSH45* overexpression lines exhibited significantly higher shoot total P concentration than the WT, whereas *osh45* showed no significant difference with WT (**Figure 3B**). In addition, no significant differences were observed in shoot concentrations of K, Fe, Ca, or Cu among WT, *osh45* and *OSH45* overexpression lines (**Figures 3C–F**). These results demonstrate that OSH45 specifically regulates Pi acquisition but does not alter the uptake of other essential mineral nutrients.

### 3.4 OSH45 regulates the expression of Pi starvation responsive genes

**Figure 4:**
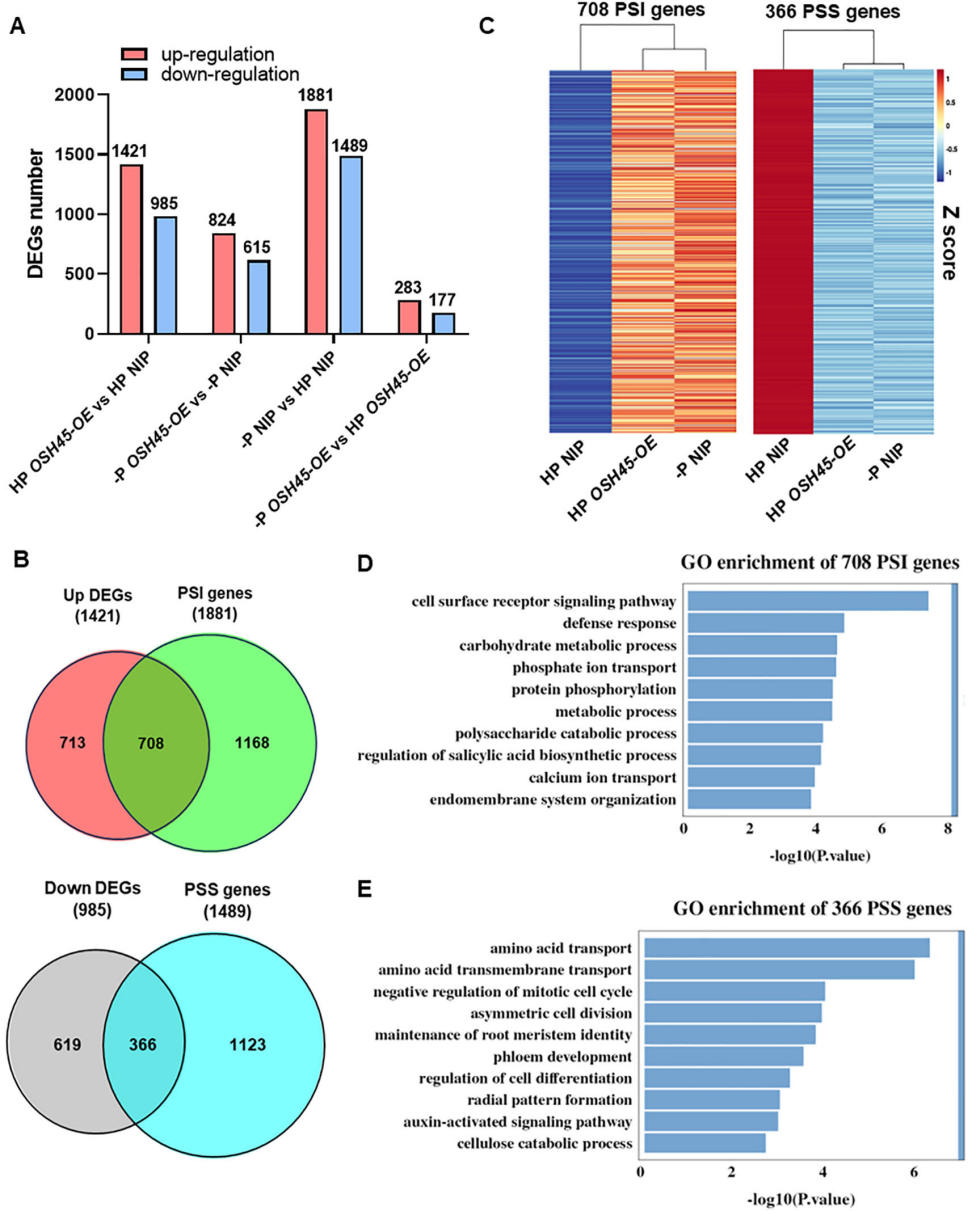
OSH45 regulates the expression of a series of phosphate starvation responsive (PSR) genes. **(A)** Numbers of differentially expressed genes (DEGs) in *OSH45-OE* lines relative to wild-type (WT) seedlings grown under high-Pi (HP, 200 μM Pi) or no-Pi (-P, 0 μM Pi) conditions. DEGs were defined as fold-change (FC) ≥ 2 and *P*-value < 0.05. Seedlings were cultured under HP conditions for 7 d and then transferred to HP or -P conditions for another 7 d before sampling and subsequent RNA-seq. **(B)** Venn diagrams showing the number of overlap DEGs in *OSH45-OE* compared to WT under HP conditions and the Pi starvation-induced (PSI) genes or the Pi starvation-suppressed (PSS) genes in WT. **(C)** Heatmaps showing the normalized expression levels of OSH45-affected 708 PSI genes and 366 PSS genes. Z-scores: red represents higher transcript levels, and blue represents lower transcript levels. **(D, E)** GO analysis of OSH45-affected 706 PSI genes **(D)** and 366 PSS genes **(E)**. Bars represent *P*-values.

To understand whether OSH45 modulates the expression of PSR genes, a comparative transcriptome analysis was performed using the WT and *OSH45* overexpression lines grown under HP or –P (0 μM Pi) conditions. There were 2,406 and 1,439 DEGs (fold-change [FC] ≥ 2, *P*-value < 0.05) in *OSH45* overexpression plants compared to WT plants under HP and –P conditions, respectively (**Figure 4A**; **Supplementary Table S1**). Approximately 60% of DEGs were upregulated in *OSH45* overexpression lines compared to the WT under both HP and –P conditions. Venn analysis indicated that 415 DEGs were uniformly changed in *OSH45* overexpression line (*OSH45*-*OE*) versus WT under both HP and –P conditions (**Supplementary Figure S6A**). These uniformly changed DEGs were significantly enriched in ‘regulation of defense response’, ‘regulation of DNA-templated transcription’, ‘regulation of jasmonic acid mediated signaling pathway’, ‘nitrate assimilation’, etc. (**Supplementary Figure S6C**). However, most DEGs identified in *OSH45* overexpression line versus WT under HP conditions (1,991) were not differentially expressed in *OSH45* overexpression line compared to WT under –P conditions. The HP-specific DEGs were enriched in functional categories including ‘secondary cell wall biogenesis’, ‘carbohydrate metabolic process’, ‘protein phosphorylation’, ‘phosphate ion transport’, etc. (**Supplementary Figure S6B**). Collectively, these findings indicated that overexpression of *OSH45* broadly affected gene expression, but the magnitude of this transcriptional reprogramming is attenuated under Pi-deficient conditions. To further dissect *OSH45-*mediated PSR, transcriptomic profiles of the WT and *OSH45* overexpression lines under HP and –P conditions were compared. Strikingly, there were 3,370 DEGs between –P and HP treatments in WT, including 1,881 phosphate starvation induced (PSI) genes and 1,489 phosphate starvation suppressed (PSS) genes. In contrast, only 460 DEGs between –P and HP treatments were detected in the *OSH45* overexpression line, including 283 PSI genes and 177 PSS genes (**Figure 4A**). While 97% (3,274 out of 3,370) of the PSR genes were not responsive to Pi starvation in the *OSH45-OE* line, only 96 common PSR genes were found between the *OSH45-OE* line and the WT (**Supplementary Figure S7A**). These 3,274 genes were significantly enriched in the following GO terms: ‘hydrogen peroxide catabolic process’, ‘cellular oxidant detoxification’, ‘response to oxidative stress’, ‘carbohydrate metabolic process’, and ‘phosphate ion transport’, etc. (**Supplementary Figure S7B**). It suggests that *OSH45* overexpression decreased the sensitivity to Pi starvation. By comparing the PSR genes in WT (–P vs HP) and the DEGs in *OSH45* overexpression lines vs WT under Pi sufficiency, we found that the expression of approximately 38% of the PSI genes (708/1881) were upregulated and about 25% of the PSS genes (366/1489) were down-regulated in *OSH45* overexpression line under Pi-sufficient conditions (**Figure 4B**; **Supplementary Table S2**). Heatmap analysis revealed that the expression of the 708 PSI genes was upregulated in WT under Pi-deficient conditions and in the *OSH45* overexpression lines compared to the WT under HP conditions (**Figure 4C**). Conversely, the 366 PSS genes were downregulated in WT under Pi starvation and in the *OSH45* overexpression lines compared to the WT under HP conditions (**Figure 4C**). GO analysis showed that the 708 PSI genes upregulated in *OSH45-OE* plants and –P WT are significantly enriched in terms ‘cell surface receptor signaling pathway’, ‘defense response’, ‘carbohydrate metabolic process’, ‘phosphate ion transport’, ‘protein phosphorylation’, etc. (**Figure 4D**; **Supplementary Table S3**). In contrast, the 366 PSS genes downregulated in *OSH45* overexpression lines and –P WT were enriched in GO term including ‘amino acid transport’, ‘negative regulation of mitotic cell cycle’, ‘asymmetric cell division’ and ‘maintenance of root meristem identify’, etc. (**Figure 4E**; **Supplementary Table S3**). All these results suggest that overexpression of *OSH45* constitutively activates PSR.

### 3.5 Overexpression of *OSH45* affected the expression of a series of phosphate transporters

**Figure 5:**
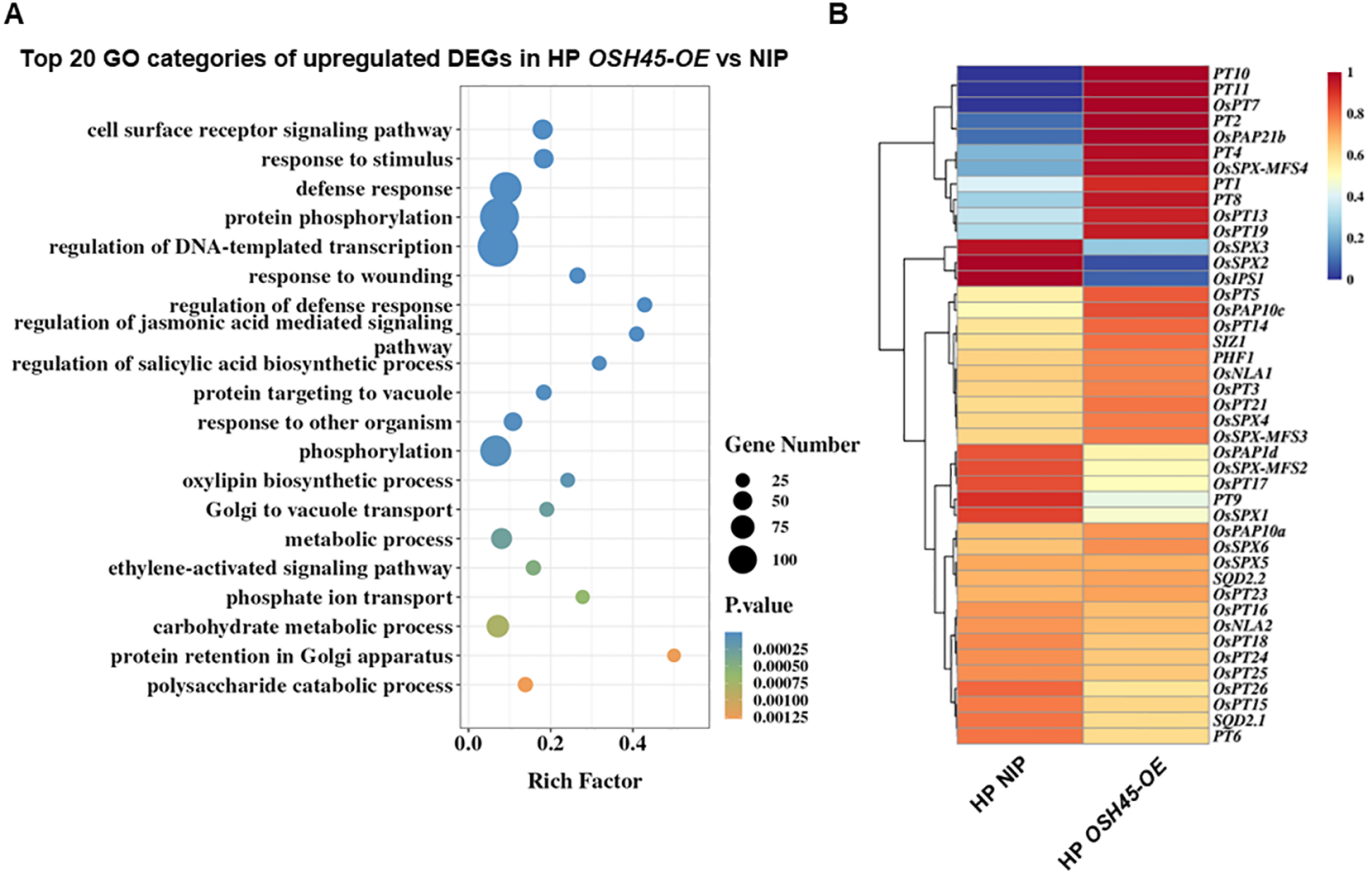
Biological processes of upregulated DEGs in *OSH45-OE* vs NIP and the expression of known Pi signaling related genes in *OSH45-OE* and NIP. **(A)** Gene ontology (GO) analysis of upregulated DEGs in *OSH45* overexpression lines vs NIP under HP conditions. The top twenty significantly enriched GO terms (*P*-values for biological processes) are shown. **(B)** Heatmaps showing the normalized expression levels of OSH45-affected Pi signaling and transport related genes. Red represents higher transcript levels, and blue represents lower transcript levels.

**Figure 6:**
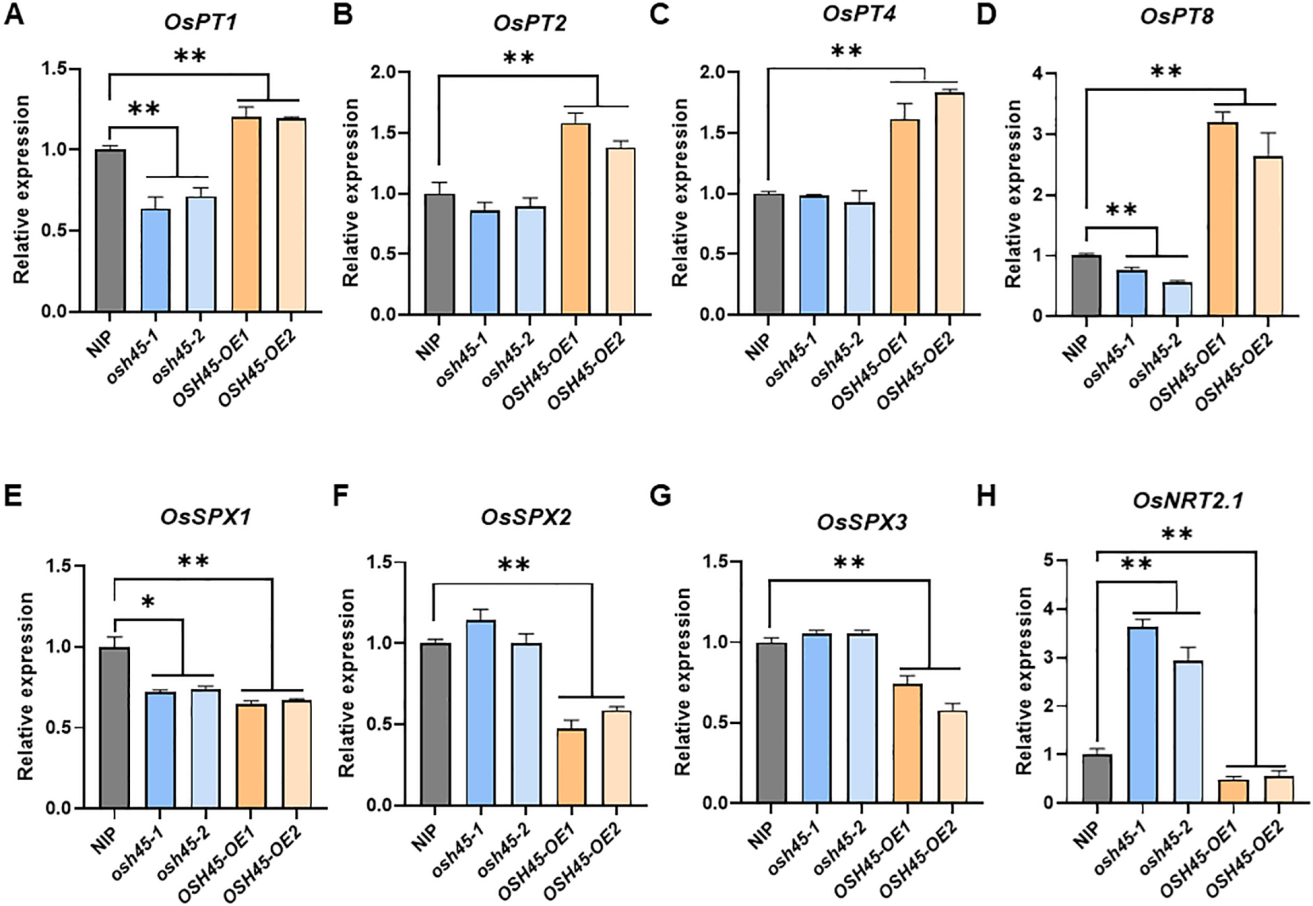
RT-qPCR analysis of the expression levels of *OsPT1* **(A)**, *OsPT2* **(B)**, *OsPT4* **(C)**, *OsPT8* **(D)**, *OsSPX1* **(E)**, *OsSPX2* **(F)**, *OsSPX3* **(G)** and *OsNRT2.1* **(H)** in NIP, *osh45* mutants, and *OSH45* overexpression plants cultured under HP conditions. *ACTIN1* was used as an endogenous control. The expression level of each gene in the wild type NIP was set to 1. Data are means ± SD (*n* = 3); asterisks indicate significant differences compared to wild-type NIP (*P < 0.05; **P < 0.01; Student’s *t-*test).

To delineate OSH45-regulated biological processes, we analyzed the DEGs of *OSH45* overexpression line versus WT under Pi-sufficient conditions. The 1,421 upregulated DEGs in *OSH45* overexpression line were enriched in GO terms ‘defense response’, ‘protein phosphorylation’, ‘regulation of DNA-templated transcription’, and ‘phosphate ion transport’, etc. (**Figure 5A**; **Supplementary Table S4**). The enriched term ‘phosphate ion transport’ in the upregulated DEGs suggests that OSH45 may be an important transcriptional regulator orchestrating Pi acquisition. In addition, the 985 downregulated DEGs in *OSH45* overexpression plants were enriched in biological processes ‘plant-type secondary cell wall biogenesis’, ‘amino acid transport’, ‘hydrogen peroxide catabolic process’, ‘nitric oxide biosynthetic process’ and ‘nitrate assimilation’, etc. (**Supplementary Figure S8**; **Supplementary Table S4**). The enrichment of terms ‘amino acid transport’ and ‘nitrate assimilation’ suggests that OSH45 may exert a negative regulatory role in nitrogen assimilation pathways. We further analyzed the expression of known Pi signaling- or homeostasis-related genes, in the *OSH45* overexpression lines. Among the known Pi signaling- or homeostasis-related genes, the transcript levels of phosphate transporters (*OsPT1, PT2, PT4, PT8, PT10* and *PT11*), which are involved in Pi uptake from the soil, and the transcript levels of purple acid phosphatase *OsPAP21b* and *OsPAP10c* which are involved in Pi release from organic P, were increased in roots of *OSH45* overexpression lines; whereas the transcript abundances of *OsSPX1*, *OsSPX2* and *OsSPX3*, which are Pi starvation signaling suppressors, were decreased in *OSH45* overexpression lines (**Figure 5B**). To verify the RNA-seq data and to investigate whether OSH45 directly regulates Pi uptake, we analyzed the expression of several phosphate transporter genes, SPX genes, and PHR genes in *OSH45* overexpression lines and *osh45* plants by RT-qPCR. The results showed that the transcript levels of phosphate transporters *OsPT1*, *OsPT2*, *OsPT4*, and *OsPT8* were significantly upregulated, while *OsSPX1*, *OsSPX2*, and *OsSPX3* were markedly downregulated in *OSH45* overexpression plants compared to WT (**Figures 6A–G**). However, the expression of core Pi signaling regulators *OsPHR1*, *OsPHR2*, and *OsPHR4* remained unchanged in both *OSH45-OE* and *osh45* compared with WT (**Supplementary Figures S9A–D**). In *osh45*, the expression of *OsPT1* and *OsPT8* was decreased, while the transcript levels of *OsPT2*, *OsPT4*, and *OsSPX1-3* remained unchanged compared to the WT (**Figures 6A–G**). We also analyzed the expression of these genes in WT, *OSH45-OE*, and *osh45* plants under LP conditions. The expression of *OsPT1*, *OsPT2*, *OsPT4*, and *OsPT8* remained significantly upregulated, whereas the expression of *OsSPX1-3* were consistently decreased in *OSH45-OE* plants (**Supplementary Figures S10A–G**). In contrast, the expression of *OsPT1*, *OsPT2*, and *OsPT8* were decreased in *osh45* plants, while the expression levels of *OsPT4* and *OsSPX2-3* showed no significant change in *osh45* compared to WT (**Supplementary Figures S10A–G**). These results confirm that OSH45 functions as a positive regulator, activating PTs while suppressing SPX genes. It is notable that the expression level of *OsIPS1*, a LP-induced long non-coding RNA targeting *OsPHO2*, was significantly lower in *osh45* compared with the WT (**Supplementary Figures S9E, F**). These observations suggest that OSH45 participate in the regulation of Pi homeostasis. Additionally, the transcript level of nitrate transporter *OsNRT2.1* was upregulated in *osh45* but suppressed in *OSH45* overexpression line (**Figure 6H**), suggesting an antagonistic regulation of nitrogen uptake genes.

## 4 Discussion

### 4.1 OSH45 modulates PSR through transcriptional regulation of PT and SPX genes

P is an essential macronutrient for plant growth and development. To adapt to Pi-limited environments, plants have evolved systemic adaptive strategies involving transcriptional reprogramming to improve Pi acquisition and utilization. Previous studies have demonstrated that several transcription factors, such as *OsPHRs*, *OsPTF1*, and *OsWRKY74*, are involved in plant adaptation to Pi deficiency (Yi et al., 2005; Ruan et al., 2016; Dai et al., 2016; Guo et al., 2015). In this study, we identified *OSH45* as a novel transcription factor, orchestrating PSR in rice. The expression of *OSH45* is upregulated under Pi-deficient conditions (**Figure 1A**; **Supplementary Figure S1**). Transgenic plants overexpressing *OSH45* accumulated significantly higher inorganic Pi and total P in shoots compared to WT plants under Pi-sufficient conditions (**Figures 3A, B**; **Supplementary Figure S5**). Consistently, the transcript levels of PT genes *OsPT1*, *OsPT2*, *OsPT4* and *OsPT8* were elevated in *OSH45-OE* lines (**Figures 6A–D**), facilitating Pi uptake from the soil. Conversely, *OSH45* loss-of-function mutants exhibited reduced leaf and shoot Pi concentrations compared to WT (**Figures 3A**; **Supplementary Figure S5A**), consistent with a concomitant decrease in *OsPT1* and *OsPT8* transcript levels (**Figures 6A, D**). Global transcriptomic profiling revealed that overexpression of *OSH45* perturbed the expression of 32% of the PSR genes (**Figure 4B**; **Supplementary Table S3**), highlighting the important role of OSH45 in the regulation of PSR. Consistently, *OSH45* overexpression plants showed markedly improved tolerance to Pi deprivation, as evidenced by increased shoot and root biomass and greater plant height under LP conditions compared to WT (**Figure 2**; **Supplementary Figure S4**). Collectively, these enhancements in growth under Pi starvation conditions suggest that OSH45 is a potential candidate for engineering crops with improved Pi uptake and use efficiency on Pi-limited soils.

### 4.2 OSH45 might regulate LP adaptation via SPX-PHR module

Under Pi-limited conditions, plants undergo transcriptional reprogramming to upregulate key functional genes, including Pi signaling components such as *IPS1*, Pi transporters (*OsPT1*-*OsPT11*), Pi remobilization enzymes (e.g., acid phosphatase-encoding genes), and intracellular Pi partitioning mediators (*OsSPX-MFSs*, *OsVPE1/2*), etc. These adaptive responses are mainly regulated by the master regulator OsPHR2 and its homologs (Lu et al., 2022; Wang et al., 2018), while the function of OsPHR2 is negatively regulated by SPX proteins (OsSPX1–OsSPX6) (Prathap et al., 2022). Transcriptome analysis revealed that about 38% of the PSI genes were upregulated and 25% of the PSS genes were downregulated in the *OSH45* overexpression line (**Figure 4B**), suggesting that OSH45 is an important regulator of PSR genes. Considering that the central regulatory role of OsPHR2 in PSR genes expression and the phenotypic similarity in Pi hyperaccumulation between *OSH45-* and *OsPHR2* overexpression lines, we hypothesize that OSH45 affects OsPHR2 function and thereby modulates the expression of PSR genes. Transcriptomic data and RT-qPCR analyses revealed that there were no significant changes in *OsPHR2* transcript levels in either *OSH45* knockout mutants or overexpression lines compared to WT plants (**Supplementary Figure S9**), indicating that OSH45 does not regulate the expression of *OsPHR2*. Intriguingly, the expression of key Pi signaling negative regulators *OsSPX1*, *OsSPX2*, and *OsSPX3* were markedly downregulated in *OSH45* overexpression lines (**Figures 6E–G**). This result suggests that OSH45 may enhance OsPHR2 function through downregulating the expression of *OsSPXs*, which inhibiting OsPHR2 function.

### 4.3 OSH45 may modulate P and nitrogen-associated transcriptional responses and plant development

The above results indicate that OSH45 positively regulates a series of PSR genes, including phosphate transporter genes. In parallel, GO analysis of the downregulated DEGs in *OSH45* overexpression line (*OSH45-OE vs* NIP) revealed significant enrichment in categories related to “nitrate assimilation” and “amino acid transport” (**Supplementary Figure S8**). Specifically, the transcript levels of the high-affinity nitrate transporter *OsNRT2.1* and nitrate reductase *OsNIA1* were significantly reduced in *OSH45-OE* compared to WT (**Figure 6H**; **Supplementary Table S2**). These findings suggest that OSH45 may repress nitrogen uptake and assimilation. This repression is consistent with previous studies showing that Pi starvation alters nitrogen metabolism. In particular, SPX-PHR module and NIGT1 have been shown to integrate Pi and nitrate signaling, repressing *NRT* genes expression under LP conditions (Hu et al., 2019; Wang et al., 2020c; Ueda et al., 2020; Wang et al., 2023a). The simultaneous upregulation of Pi acquisition genes and downregulation of nitrate-associated genes in *OSH45-OE* lines may reflect a broader regulatory mechanism in maintaining nutrient balance under Pi starvation. In addition, *OSH45* overexpression activated both the phosphate ion transport and plant defense response pathways (**Figure 5A**). This result implies potential crosstalk between nutrient signaling and immune pathways. Recently, OsSPX1 and OsSPX2 have been shown to fine-tune brassinosteroid signaling to balance growth and immunity depending on Pi availability (He et al., 2023). The dual regulation of Pi uptake and defense-related genes suggests that OSH45 may contribute to the coordination of nutrient status and stress responses to enhance plant fitness in nutrient-limited environments.

In summary, we characterized OSH45 as a positive regulator of PSR. Our data showed that *OSH45* positively regulated Pi acquisition and plant growth under LP conditions, whereas *OSH45* negatively regulated N acquisition and assimilation related genes. Our results expand the knowledge of molecular regulatory networks underlying plant adaptation to nutrient limitation and identify potential target for P-efficient crop breeding.

## Data Availability

The datasets presented in this study can be found in online repositories. The names of the repository/repositories and accession number(s) can be found below: https://ngdc.cncb.ac.cn/gsa. Accession No. CRA026415.

## References

[B1] Brinch-Pedersen H. Sørensen L. D. Holm P. B. (2002). Engineering crop plants: getting a handle on phosphate. Trends Plant Sci. 7, 118–125. doi: 10.1016/S1360-1385(01)02222-1, PMID: 11906835

[B2] Bustos R. Castrillo G. Linhares F. Puga M. I. Rubio V. Perez-Perez J.. (2010). A central regulatory system largely controls transcriptional activation and repression responses to phosphate starvation in Arabidopsis. PloS Genet. 6, e1001102. doi: 10.1371/journal.pgen.1001102, PMID: 20838596 PMC2936532

[B3] Chen C. Chen H. Zhang Y. Thomas H. R. Frank M. H. He Y.. (2020). TBtools: an integrative toolkit developed for interactive analyses of big biological data. Mol. Plant 13, 1194–1202. doi: 10.1016/j.molp.2020.06.009, PMID: 32585190

[B4] Chen J. Wang Y. Wang F. Yang J. Gao M. Li C.. (2015). The rice CK2 kinase regulates trafficking of phosphate transporters in response to phosphate levels. Plant Cell 27, 711–723. doi: 10.1105/tpc.114.135335, PMID: 25724641 PMC4558666

[B5] Chen Y. Qi H. Yang L. Xu L. Wang J. Guo J.. (2023). The OsbHLH002/OsICE1-OSH1 module orchestrates secondary cell wall formation in rice. Cell Rep. 42, 112702. doi: 10.1016/j.celrep.2023.112702, PMID: 37384532

[B6] Chiou T. J. Lin S. I. (2011). Signaling network in sensing phosphate availability in plants. Annu. Rev. Plant Biol. 62, 185–206. doi: 10.1146/annurev-arplant-042110-103849, PMID: 21370979

[B7] Dai X. Wang Y. Yang A. Zhang W.-H. (2012). OsMYB2P-1, an R2R3 MYB transcription factor, is involved in the regulation of phosphate-starvation responses and root architecture in rice. Plant Physiol. 159, 169–183. doi: 10.1104/pp.112.194217, PMID: 22395576 PMC3375959

[B8] Dai X. Wang Y. Zhang W. H. (2016). OsWRKY74, a WRKY transcription factor, modulates tolerance to phosphate starvation in rice. J. Exp. Bot. 67, 947–960. doi: 10.1093/jxb/erv515, PMID: 26663563 PMC4737085

[B9] Devaiah B. N. Karthikeyan A. S. Raghothama K. G. (2007). WRKY75 transcription factor is a modulator of phosphate acquisition and root development in Arabidopsis. Plant Physiol. 143, 1789–1801. doi: 10.1104/pp.106.093971, PMID: 17322336 PMC1851818

[B10] Furumizu C. Alvarez J. P. Sakakibara K. Bowman J. L. (2015). Antagonistic roles for KNOX1 and KNOX2 genes in patterning the land plant body plan following an ancient gene duplication. PloS Genet. 11, e1004980. doi: 10.1371/journal.pgen.1004980, PMID: 25671434 PMC4335488

[B11] Guan Z. Zhang Q. Zhang Z. Zuo J. Chen J. Liu R.. (2022). Mechanistic insights into the regulation of plant phosphate homeostasis by the rice SPX2 - PHR2 complex. Nat. Commun. 13, 1581. doi: 10.1038/s41467-022-29275-8, PMID: 35332155 PMC8948245

[B12] Guo M. Ruan W. Li C. Huang F. Zeng M. Liu Y.. (2015). Integrative comparison of the role of the PHOSPHATE RESPONSE1 subfamily in phosphate signaling and homeostasis in rice. Plant Physiol. 168, 1762–1776. doi: 10.1104/pp.15.00736, PMID: 26082401 PMC4528768

[B13] Hake S. Smith H. M. S. Holtan H. Magnani E. Mele G. Ramirez J. (2004). The role of KNOX genes in plant development. Annu. Rev. Cell Dev. Biol. 20, 125–151. doi: 10.1146/annurev.cellbio.20.031803.093824, PMID: 15473837

[B14] Ham B. K. Chen J. Yan Y. Lucas W. J. (2018). Insights into plant phosphate sensing and signaling. Curr. Opin. Biotechnol. 49, 1–9. doi: 10.1016/j.copbio.2017.07.005, PMID: 28732264

[B15] He Q. Lu H. Guo H. Wang Y. Zhao P. Li Y.. (2021). OsbHLH6 interacts with OsSPX4 and regulates the phosphate starvation response in rice. Plant J. 105, 649–667. doi: 10.1111/tpj.15061, PMID: 33128314

[B16] He Y. Zhao Y. Hu J. Wang L. Li L. Zhang X.. (2023). The OsBZR1–OsSPX1/2 module fine-tunes the growth–immunity trade-off in adaptation to phosphate availability in rice. Mol. Plant 17, 258–276. doi: 10.1016/j.molp.2023.12.003, PMID: 38069474

[B17] Hu B. Jiang Z. Wang W. Qiu Y. Zhang Z. Liu Y.. (2019). Nitrate-NRT1.1B-SPX4 cascade integrates nitrogen and phosphorus signalling networks in plants. Nat. Plants 5, 401–413. doi: 10.1038/s41477-019-0384-1, PMID: 30911122

[B18] Johnston A. E. Poulton P. R. Fixen P. E. Curtin D. (2014). Phosphorus: its efficient use in agriculture. Adv. Agron. 123, 177–228. doi: 10.1016/B978-0-12-420225-2.00005-4

[B19] Liu S. Xu Z. Essemine J. Liu Y. Liu C. Zhang F.. (2024). GWAS unravels acid phosphatase ACP2 as a photosynthesis regulator under phosphate starvation conditions through modulating serine metabolism in rice. Plant Commun. 5, e100885. doi: 10.1016/j.xplc.2024.100885, PMID: 38504521 PMC11287135

[B20] Lu H. Wang F. Wang Y. Lin R. Wang Z. Mao C. (2022). Molecular mechanisms and genetic improvement of low-phosphorus tolerance in rice. Plant Cell Environment. 46 (4), 1104–1119. doi: 10.1111/pce.14457, PMID: 36208118

[B21] Lv Q. Zhong Y. Wang Y. Wang Z. Zhang L. Shi J.. (2014). SPX4 negatively regulates phosphate signaling and homeostasis through its interaction with PHR2 in rice. Plant Cell 26, 1586–1597. doi: 10.1105/tpc.114.123208, PMID: 24692424 PMC4036573

[B22] Lynch J. P. (2011). Root phenes for enhanced soil exploration and phosphorus acquisition: tools for future crops. Plant Physiol. 156, 1041–1049. doi: 10.1104/pp.111.175414, PMID: 21610180 PMC3135935

[B23] Ma X. Zhang Q. Zhu Q. Liu W. Chen Y. Qiu R.. (2015). A robust CRISPR/cas9 system for convenient, high-efficiency multiplex genome editing in monocot and dicot plants. Mol. Plant 8, 1274–1284. doi: 10.1016/j.molp.2015.04.007, PMID: 25917172

[B24] Madison I. Gillan L. Peace J. Gabrieli F. Van Den Broeck L. Jones J. L.. (2023). Phosphate starvation: response mechanisms and solutions. J. Exp. Bot. 74 (21), 6417–6430. doi: 10.1093/jxb/erad326, PMID: 37611151

[B25] Paz-Ares J. Puga M. I. Rojas-Triana M. Martinez-Hevia I. Diaz S. Poza-Carrion C.. (2022). Plant adaptation to low phosphorus availability: Core signaling, crosstalks, and applied implications. Mol. Plant 15, 104–124. doi: 10.1016/j.molp.2021.12.005, PMID: 34954444

[B26] Poirier Y. Jaskolowski A. Clúa J. (2022). Phosphate acquisition and metabolism in plants. Curr. Biol. 32, R623–R629. doi: 10.1016/j.cub.2022.03.073, PMID: 35728542

[B27] Prathap V. Kumar A. Maheshwari C. Tyagi A. (2022). Phosphorus homeostasis: acquisition, sensing, and long-distance signaling in plants. Mol. Biol. Rep. 49, 8071–8086. doi: 10.1007/s11033-022-07354-9, PMID: 35318578

[B28] Puga M. I. Poza-Carrión C. Martinez-Hevia I. Perez-Liens L. Paz-Ares J. (2024). Recent advances in research on phosphate starvation signaling in plants. J. Plant Res. 137, 315–330. doi: 10.1007/s10265-024-01545-0, PMID: 38668956 PMC11081996

[B29] Ruan W. Guo M. Wu P. Yi K. (2016). Phosphate starvation induced OsPHR4 mediates Pi-signaling and homeostasis in rice. Plant Mol. Biol. 93, 327–340. doi: 10.1007/s11103-016-0564-6, PMID: 27878661

[B30] Ruan W. Guo M. Xu L. Wang X. Zhao H. Wang J.. (2018). An SPX-RLI1 module regulates leaf inclination in response to phosphate availability in rice. Plant Cell 30, 853–870. doi: 10.1105/tpc.17.00738, PMID: 29610209 PMC5969273

[B31] Rubio V. Linhares F. Solano R. Martín A. C. Iglesias J. Leyva A.. (2001). A conserved MYB transcription factor involved in phosphate starvation signaling both in vascular plants and in unicellular algae. Genes Dev. 15, 2122–2133. doi: 10.1101/gad.204401, PMID: 11511543 PMC312755

[B32] Sheng M. Ma X. Wang J. Xue T. Li Z. Cao Y.. (2022). KNOX II transcription factor HOS59 functions in regulating rice grain size. Plant J. 110, 863–880. doi: 10.1111/tpj.15709, PMID: 35167131

[B33] Sun L. Song L. Zhang Y. Zheng Z. Liu D. (2016). Arabidopsis PHL2 and PHR1 act redundantly as the key components of the central regulatory system controlling transcriptional responses to phosphate starvation. Plant Physiol. 170, 499–514. doi: 10.1104/pp.15.01336, PMID: 26586833 PMC4704584

[B34] Tamaoki M. Tsugawa H. Minami E. I. Kayano T. Yamamoto N. Kano-Murakami Y.. (1995). Alternative RNA products from a rice homeobox gene. Plant J. 7, 927–938. doi: 10.1046/j.1365-313X.1995.07060927.x, PMID: 7599652

[B35] Tsuda K. Ito Y. Sato Y. Kurata N. (2011). Positive autoregulation of a KNOX gene is essential for shoot apical meristem maintenance in rice. Plant Cell 23, 4368–4381. doi: 10.1105/tpc.111.090050, PMID: 22207572 PMC3269871

[B36] Tsuda K. Kurata N. Ohyanagi H. Hake S. (2014). Genome-wide study of KNOX regulatory network reveals brassinosteroid catabolic genes important for shoot meristem function in rice. Plant Cell 26, 3488–3500. doi: 10.1105/tpc.114.129122, PMID: 25194027 PMC4213158

[B37] Ueda Y. Kiba T. Yanagisawa S. (2020). Nitrate-inducible NIGT1 proteins modulate phosphate uptake and starvation signalling via transcriptional regulation of SPX genes. Plant J. 102, 448–466. doi: 10.1111/tpj.14637, PMID: 31811679

[B38] Wang F. Deng M. Chen J. He Q. Jia X. Guo H.. (2020a). CASEIN KINASE2-dependent phosphorylation of PHOSPHATE2 fine-tunes phosphate homeostasis in rice. Plant Physiol. 183, 250–262. doi: 10.1104/pp.20.00078, PMID: 32161109 PMC7210639

[B39] Wang F. Deng M. Xu J. Zhu X. Mao C. (2018). Molecular mechanisms of phosphate transport and signaling in higher plants. Semin. Cell Dev. Biol. 74, 114–122. doi: 10.1016/j.semcdb.2017.06.013, PMID: 28648582

[B40] Wang Z. Ruan W. Shi J. Zhang L. Xiang D. Yang C.. (2014). Rice SPX1 and SPX2 inhibit phosphate starvation responses through interacting with PHR2 in a phosphate-dependent manner. Proc. Natl. Acad. Sci. United States America 111, 14953–14958. doi: 10.1073/pnas.1404680111, PMID: 25271318 PMC4205599

[B41] Wang X. Wang H. F. Chen Y. Sun M. M. Wang Y. Chen Y. F. (2020c). The transcription factor NIGT1.2 modulates both phosphate uptake and nitrate influx during phosphate starvation in Arabidopsis and Maize. Plant Cell 32, 3519–3534. doi: 10.1105/tpc.20.00361, PMID: 32958562 PMC7610294

[B42] Wang Y. Wang F. Lu H. Lin R. Liu J. Liu Y.. (2023b). Rice chromatin protein OsHMGB1 is involved in phosphate homeostasis and plant growth by affecting chromatin accessibility. New Phytologist. 240 (2), 727–743. doi: 10.1111/nph.19189, PMID: 37553956

[B43] Wang Y. Wang F. Lu H. Liu Y. Mao C. (2021). Phosphate uptake and transport in plants: An elaborate regulatory system. Plant Cell Physiol. 62, 564–572. doi: 10.1093/pcp/pcab011, PMID: 33508131

[B44] Wang F. Wang Y. Ying L. Lu H. Liu Y. Liu Y.. (2023a). Integrated transcriptomic analysis identifies coordinated responses to nitrogen and phosphate deficiency in rice. Front. Plant Sci. 14, 116441. doi: 10.3389/fpls.2023.1164441, PMID: 37223782 PMC10200874

[B45] Wang S. Xu T. Chen M. Geng L. Huang Z. Dai X.. (2022). The transcription factor OsWRKY10 inhibits phosphate uptake via suppressing OsPHT1;2 expression under phosphate-replete condition in rice. J. Exp. Bot. 74, 1074–1089. doi: 10.1093/jxb/erac456, PMID: 36402551 PMC9899414

[B46] Wang S. Yamaguchi M. Grienenberger E. Martone P. T. Samuels A. L. Mansfield S. D. (2020b). The Class II KNOX genes KNAT3 and KNAT7 work cooperatively to influence deposition of secondary cell walls that provide mechanical support to Arabidopsis stems. Plant J. 101, 293–309. doi: 10.1111/tpj.14541, PMID: 31587430

[B47] Wu K. Yang W. T. Baek D. Yun D.-J. Lee K. S. Hong S. Y.. (2018). Rice OsMYB5P improves plant phosphate acquisition by regulation of phosphate transporter. PloS One 13, e0194628. doi: 10.1371/journal.pone.0194628, PMID: 29566032 PMC5864048

[B48] Yang W. T. Baek D. Yun D.-J. Hwang W. H. Park D. S. Nam M. H.. (2014). Overexpression of OsMYB4P, an R2R3-type MYB transcriptional activator, increases phosphate acquisition in rice. Plant Physiol. Biochem. 80, 259–267. doi: 10.1016/j.plaphy.2014.02.024, PMID: 24813725

[B49] Yi K. Wu Z. Zhou J. Du L. Guo L. Wu Y.. (2005). OsPTF1, a novel transcription factor involved in tolerance to phosphate starvation in rice. Plant Physiol. 138, 2087–2096. doi: 10.1104/pp.105.063115, PMID: 16006597 PMC1183397

[B50] Yu Y. (2019). OsKNAT7 bridges secondary cell wall formation and cell growth regulation. Plant Physiol. 181, 385–386. doi: 10.1104/pp.19.01018, PMID: 31582471 PMC6776844

[B51] Zhang J. Gu M. Liang R. Shi X. Chen L. Hu X.. (2020). OsWRKY21 and OsWRKY108 function redundantly to promote phosphate accumulation through maintaining the constitutive expression of OsPHT1;1 under phosphate-replete conditions. New Phytol. 229, 1598–1614. doi: 10.1111/nph.16931, PMID: 32936937 PMC7820984

[B52] Zhang Z. Li Z. Wang W. Jiang Z. Guo L. Wang X.. (2021). Modulation of nitrate-induced phosphate response by the MYB transcription factor RLI1/HINGE1 in the nucleus. Mol. Plant 14, 517–529. doi: 10.1016/j.molp.2020.12.005, PMID: 33316467

[B53] Zhou J. Hu Q. Xiao X. Yao D. Ge S. Ye J.. (2021). Mechanism of phosphate sensing and signaling revealed by rice SPX1-PHR2 complex structure. Nat. Commun. 12, 7040. doi: 10.1038/s41467-021-27391-5, PMID: 34857773 PMC8639918

