# Supplementary material for: OSH45, a homeobox transcription factor, coordinates low-phosphate adaptation in rice
Source: Front Plant Sci. 2025 Aug 28;16:1654599. doi: 10.3389/fpls.2025.1654599 (PMC12423420; doi:10.3389/fpls.2025.1654599)
Supplement: Supplementary file 1 [file DataSheet1.pdf]

Supporting Information

Figure S1

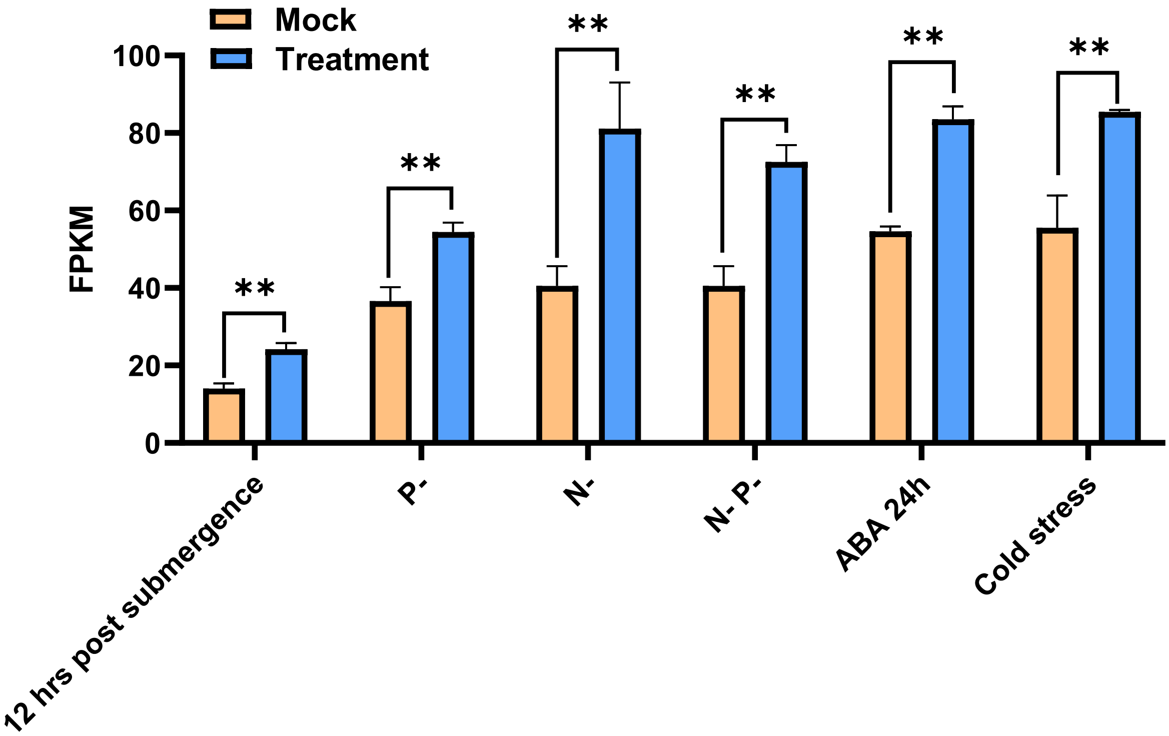

Figure S1. Expression levels of *OSH45* under different abiotic stresses. Data were from Rice RNA-seq Database (https://plantrnadb.com/ricerna/). Data are means ± SD ($n \geq 3$). **, $P < 0.01$, Student's t-test.

Figure S2

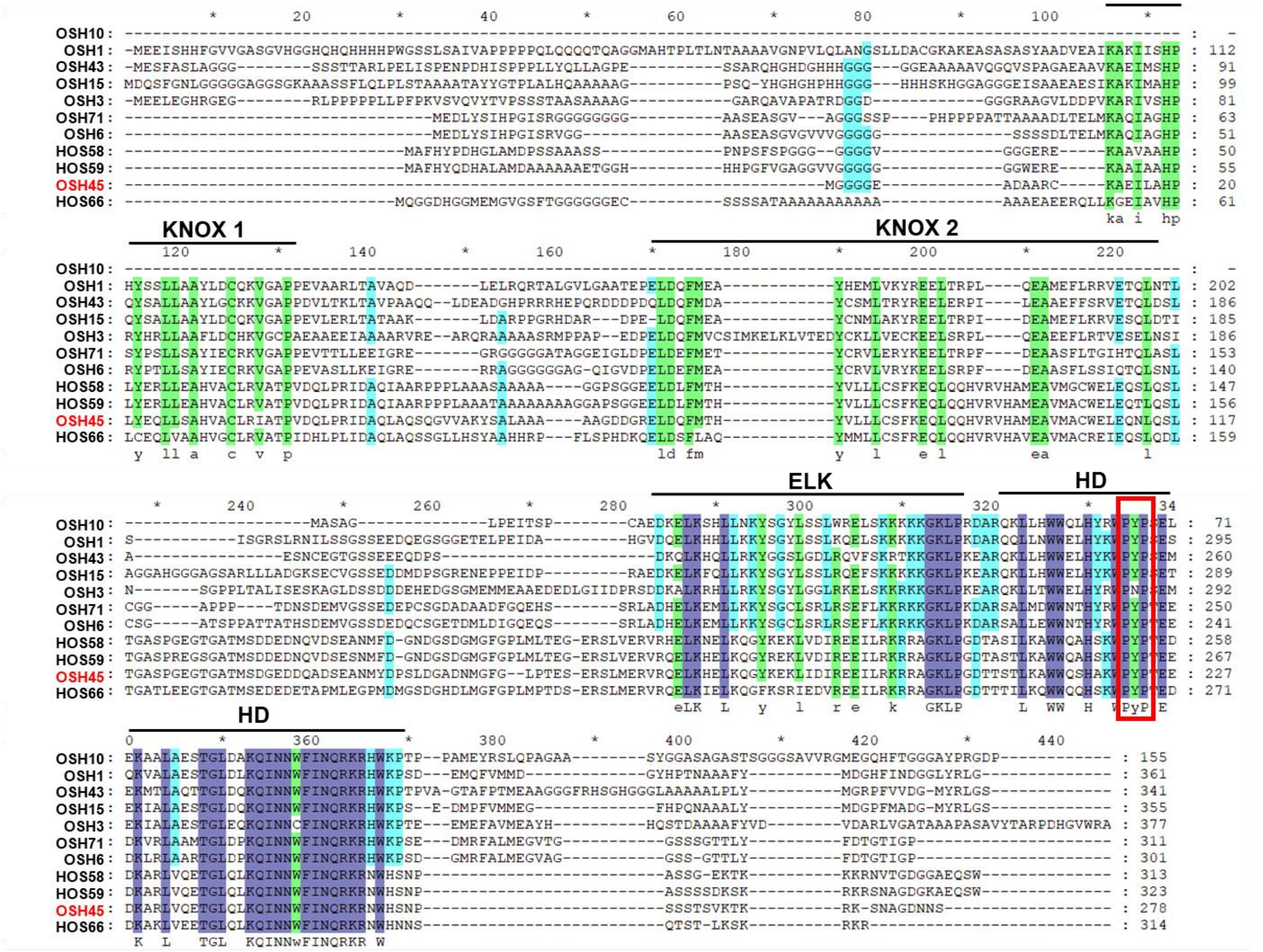

Figure S2. Sequence alignment of OSH45 and its homologous proteins in rice. Protein domains are marked with black lines. Sequence alignment was performed using GeneDoc and colored accordingly. The red box highlights the unique conserved tripeptide Proline (Pro)-Tyrosine (Tyr)-Proline (Pro) within the HD domain of KNOX family transcription factors, distinguishing it from other HD domain transcription factors.

Figure S3

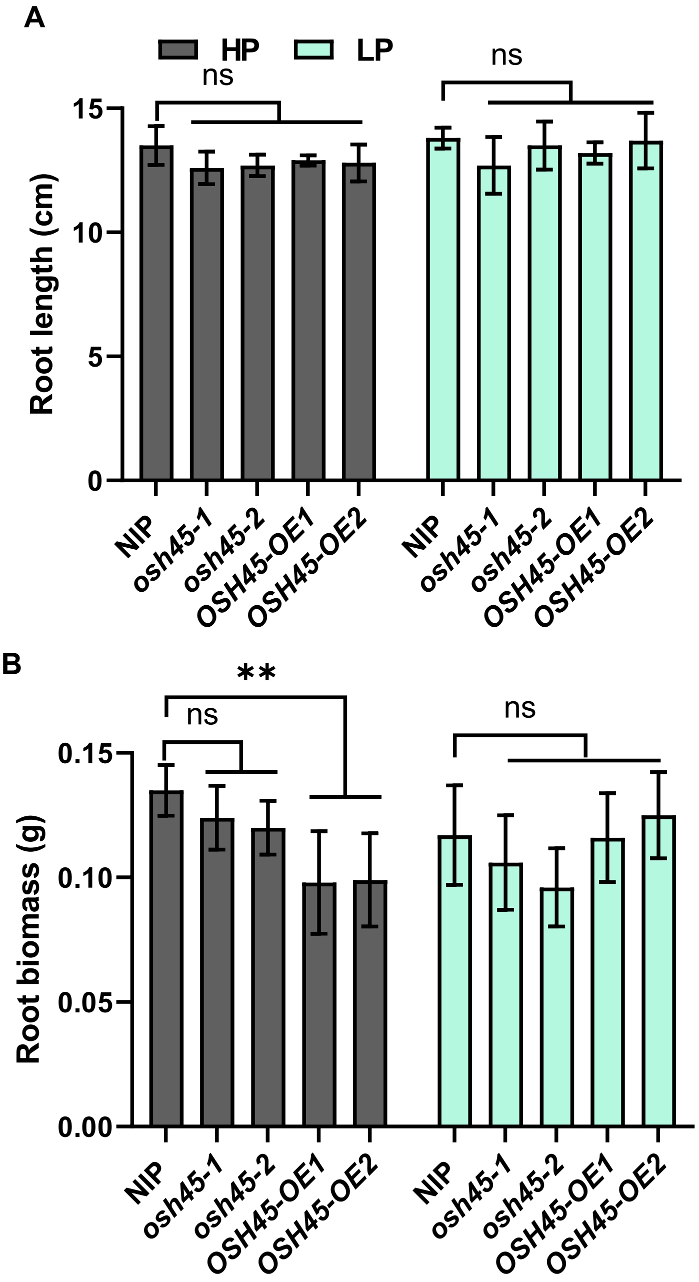

Figure S3. Root length and root biomass of *osh45* mutants and *OSH45* overexpression lines under different Pi conditions. Seven-day-old plants grown in HP (200 μM) solution were transferred to HP or LP (10 μM) conditions for another 14 d. Date are means ± SD (n = 10); ns: no significant difference, **P < 0.01; Student's t-test.

Figure S4

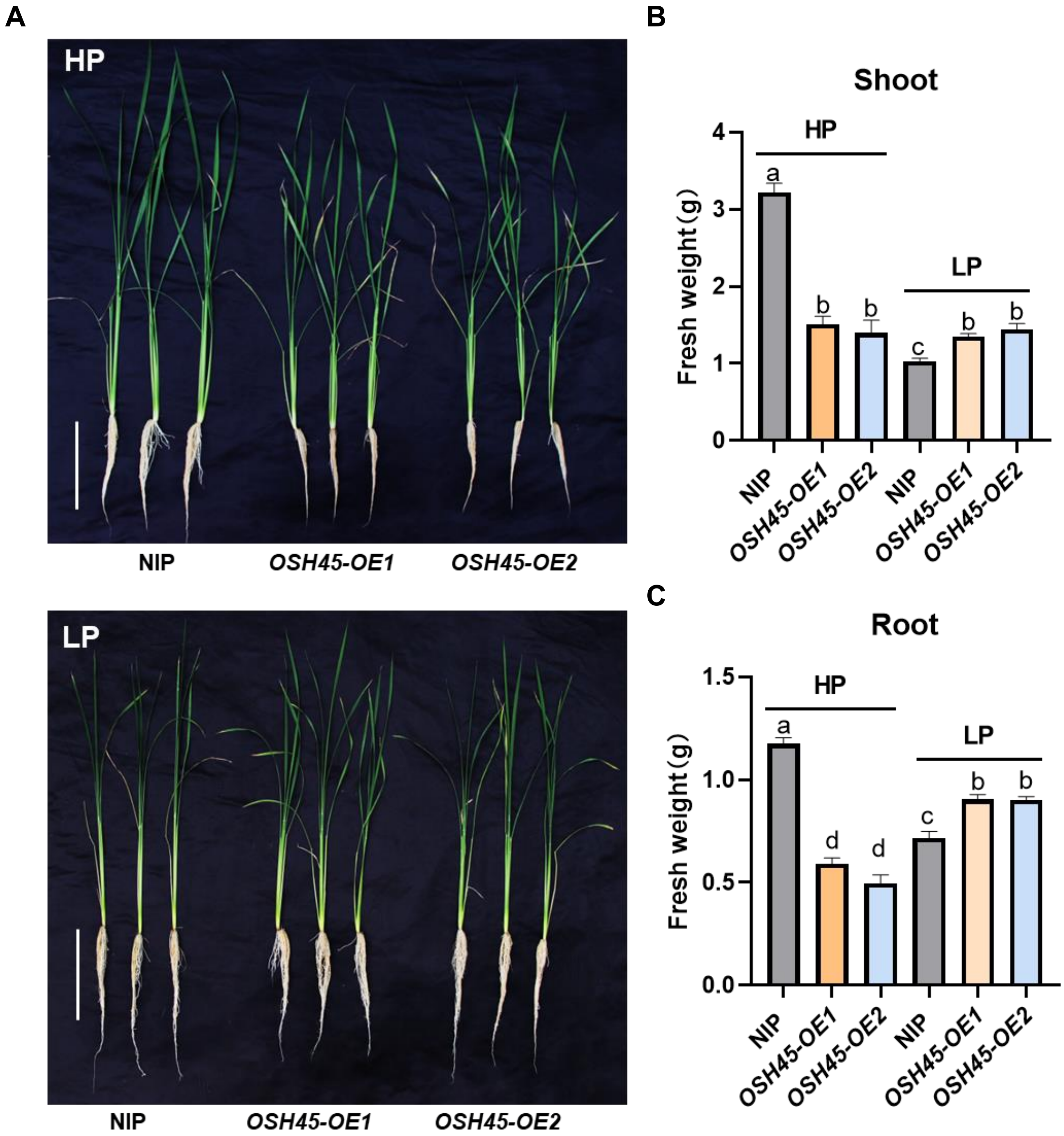

Figure S4. Phenotypes of *OSH45* overexpression lines under different Pi conditions. (A) Phenotypes of 28-day-old wild-type (NIP) and *OSH45* overexpression lines. Scale bars, 10 cm. (B) Shoot fresh weight. (C) Root fresh weight. Seven-d-old plants cultured in HP (200 μM) solution were transferred to HP or LP (10 μM) conditions and grown for another 21 d. Data are means ± SD (n = 10). Different lowercase letters indicate significant differences ($P < 0.05$; one-way ANOVA).

Figure S5

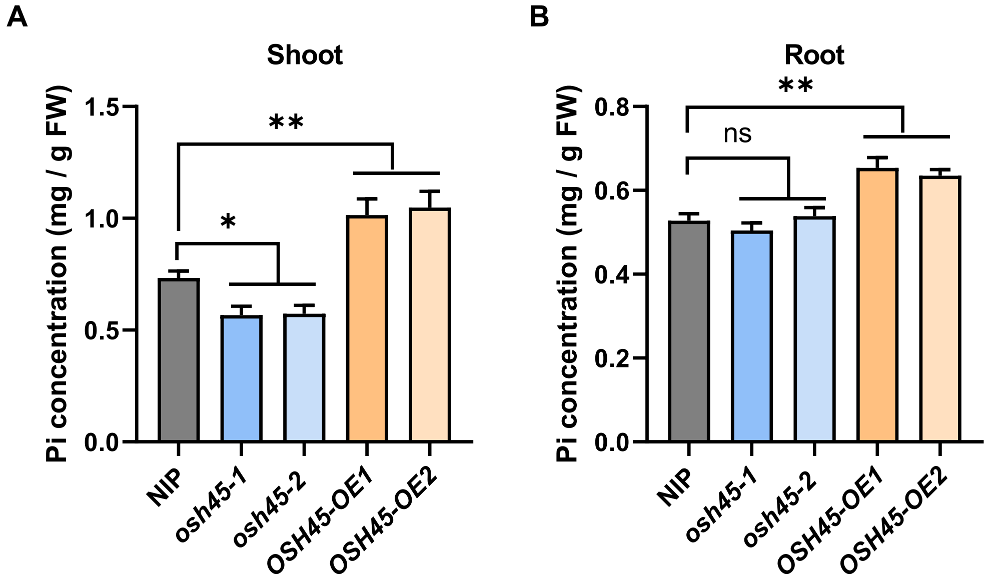

Figure S5. Pi concentration in *osh45* mutants and *OSH45* overexpression plants. (A) Shoot Pi concentration; (B) Root Pi concentration. Date are means ± SD (n=5); ns: no significant difference; *$P < 0.05$; **$P < 0.01$; Student's t-test.

Figure S6

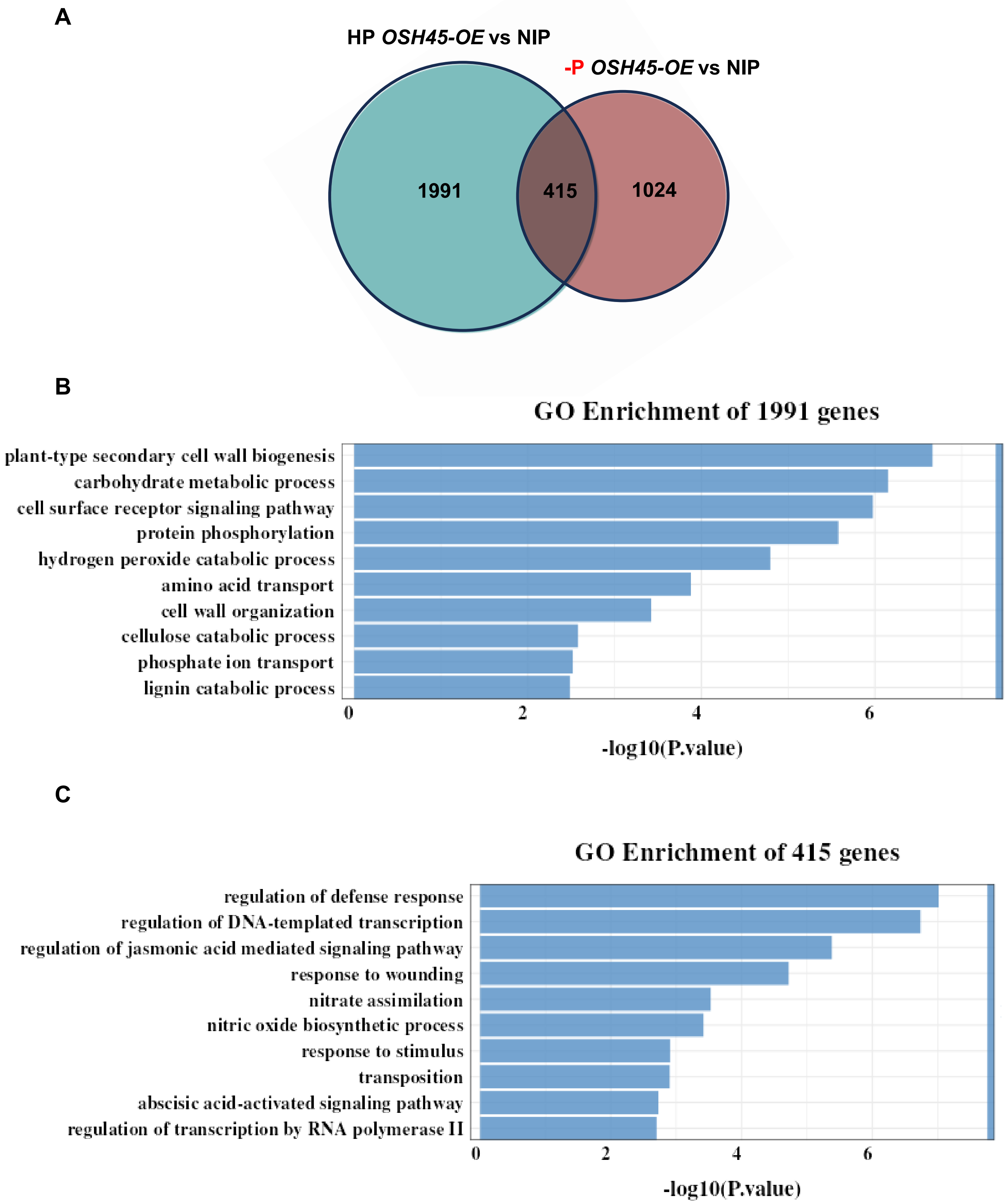

Figure S6. Transcriptomic profiling of *OSH45-OE* vs WT under HP (200 μM) and -P (0 μM) conditions. (A) Venn diagram showing the overlap of DEGs in *OSH45-OE* compared to WT under HP and -P conditions. (B, C) GO analysis for 1,991 genes regulated by OSH45 under HP (B) and the overlap 415 DEGs in HP and -P conditions (C). Bars represent enrichment significance ($-\log_{10}$ *P*-values).

Figure S7

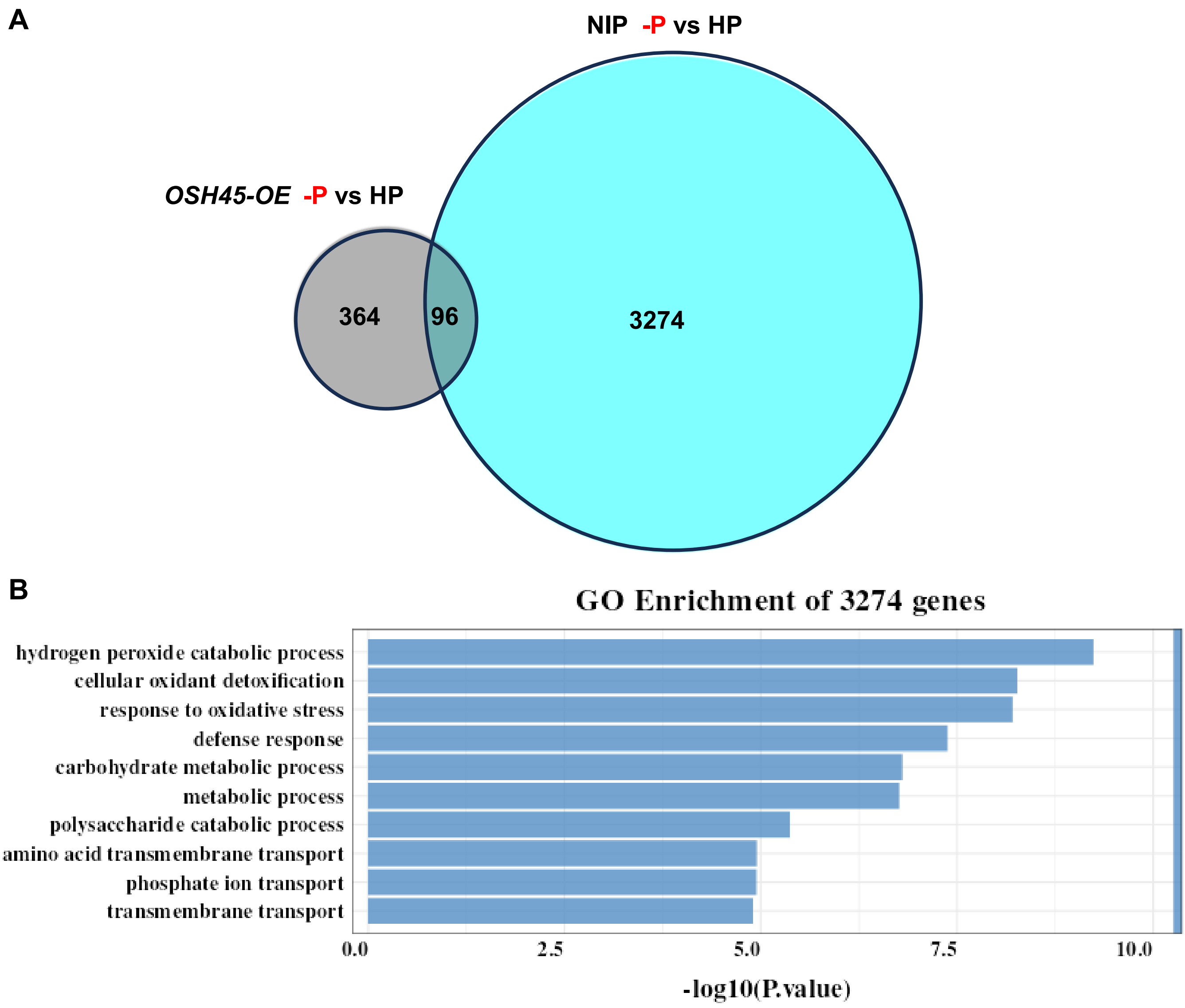

Figure S7. Transcriptome analysis of phosphate-responsive genes in *OSH45-OE* and NIP plants. (A) Venn diagram of differentially expressed genes (DEGs) in *OSH45-OE* and WT plants under high-phosphate (HP) versus no-phosphate (-P) conditions. (B) Gene Ontology (GO) analysis of 3,274 phosphate deficiency-responsive genes identified in NIP plants. Bars represent enrichment significance ($-\log_{10}$ *P*-values).

Figure S8

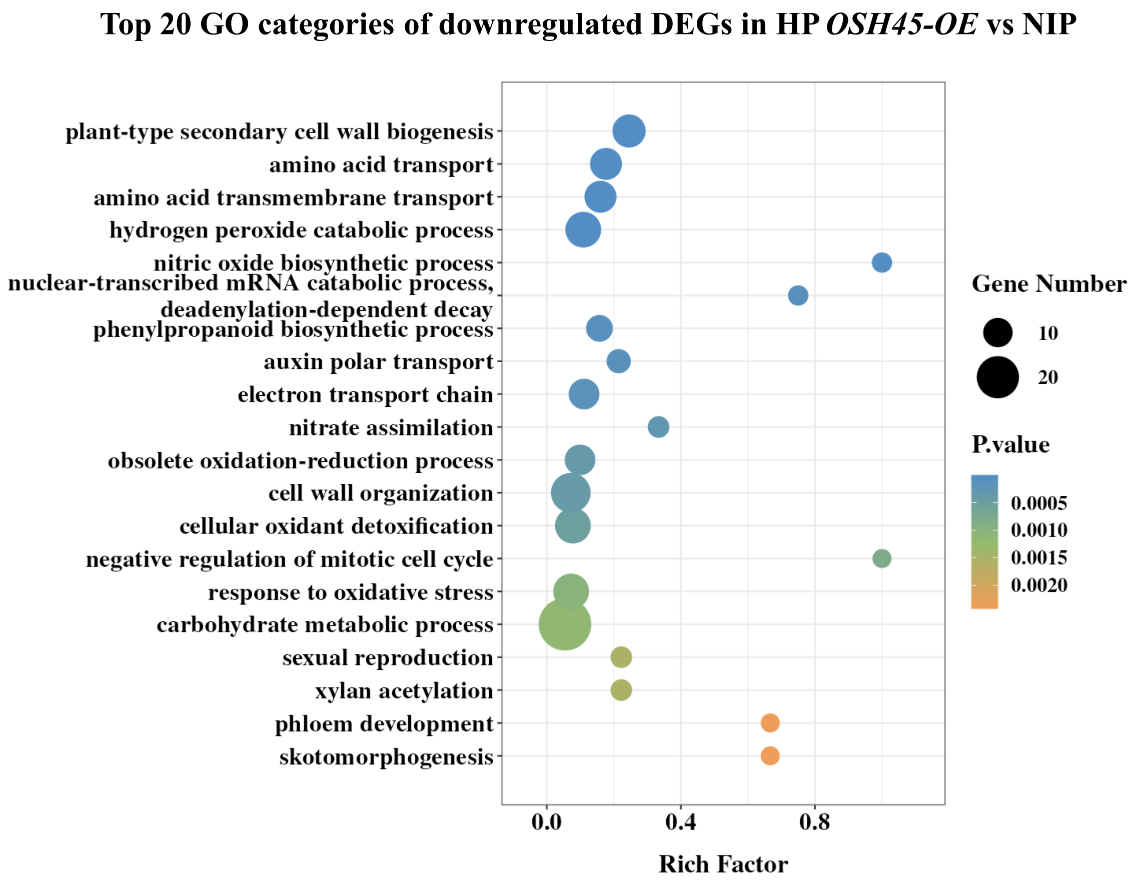

Figure S8. The top twenty significantly enriched GO terms of downregulated DEGs in *OSH45* overexpression lines relative to NIP under HP conditions.

Figure S9

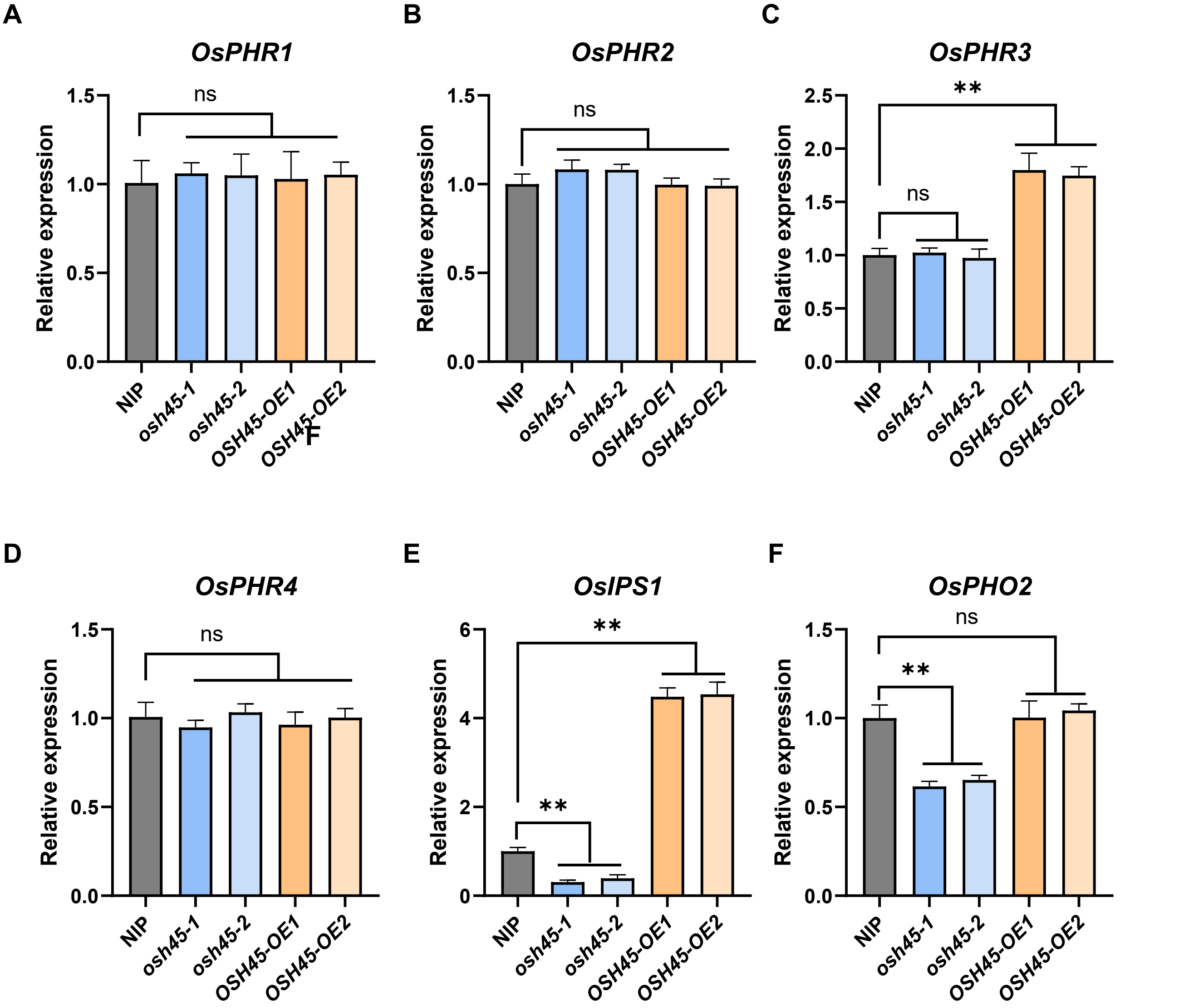

Figure S9. RT-qPCR analysis of the expression levels of *OsPHR1*, *OsPHR2*, *OsPHR3*, *OsPHR4*, *OsIPS1*, and *OsPHO2* in NIP, *osh45* mutants, and *OSH45* overexpression plants cultured under HP conditions. *ACTIN1* was used as an endogenous control. Expression of each gene in the wild type NIP was set to 1. Data are means ± SD (n = 3); ns: no significant difference; asterisks indicate significant differences compared to wild-type NIP (*, $P < 0.05$; **, $P < 0.01$; Student's *t*-test).

Figure S10

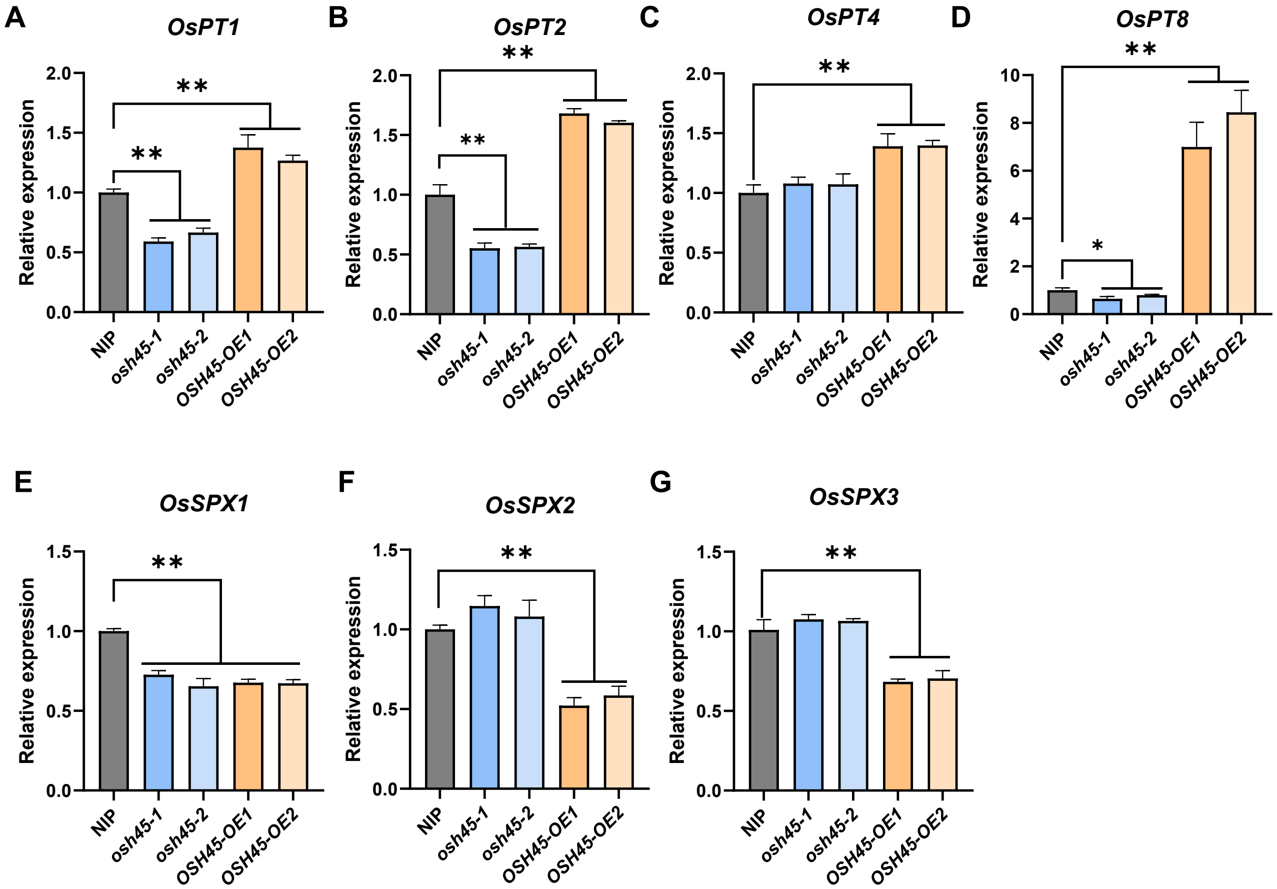

Figure S10. RT-qPCR analysis of the expression levels of *OsPT1*, *OsPT2*, *OsPT4*, *OsPT8*, *OsSPX1*, *OsSPX2* and *OsSPX3* in NIP, *osh45* mutants, and *OSH45* overexpression plants cultured under LP conditions. *ACTIN1* was used as an endogenous control. Expression of each gene in the wild type NIP was set to 1. Data are means ± SD (n = 3); asterisks indicate significant differences compared to wild-type NIP (*, $P < 0.05$; **, $P < 0.01$; Student's t-test).

Table S1. Primers used in this study

Primer	Sequence (5'-3')	Note
ACTIN-qRT-F	CAACACCCCTGCTATGTACG	For RT-qPCR
ACTIN-qRT-R	CATCACCAGAGTCCAACACAA	
OSH45-qRT-F	GGTGTAGGGACTTGCCGTTA	
OSH45-qRT-R	TTTTCTTTTGCACCCCAATG	
PT1-qRT-F	CGCTTCCGTACGAGTGGTAGT	
PT1-qRT-R	GGTTCTTTCAAATCCAGGGAAA	
PT2-qRT-F	CACAAACTTCCTCGGTATGCT	
PT2-qRT-R	GAAACCCCACAAATCCACAAC	
PT4-qRT-F	GGACATCGCCTTCTACTCGT	
PT4-qRT-R	CCCGGGAGATCTTGAACA	
PT8-qRT-F	AGAAGGCAAAAGAAATGTGTGTTAAAT	
PT8-qRT-R	AAAATGTATTCGTGCCAAATTGCT	
SPX1-qRT-F	CGACTTCCATGGCGAGAT	
SPX1-qRT-R	TCCTCTTGTCATACTTCTTGAGAATC	
SPX2-qRT-F	GCAAGAGCCTGAGCAGCCAGAT	
SPX2-qRT-R	CTCCGCCGATGCCATCTTCC	
SPX3-qRT-F	TGCAGTCCATCCGATCCG	
SPX3-qRT-R	ATGTGTATGTATGTTCTCTACCACG	
NRT2.1-qRT-F	CCTTGTTGCAAACGGTGATG	
NRT2.1-qRT-R	CCTCCGTTTGTATCGGAGAAAT	
PHR2-qRT-F	GACCAGAATTGTCTGAAGGTTCTT	
PHR2-qRT-R	ACGCAATGCCTCAGTGAGAT	
IPS1-qRT-F	TTGGCAATTATTCGGTGGAT	
IPS1-qRT-R	ACCATTTCACCATCCTCTTTATG	
PHO2-qRT-F	CGAGAATTTTGTCAAGGAGCA	
PHO2-qRT-R	TCACGAGCATGTCCAACAA	
OSH45-U3-F	GGCATGGAAGCAGTAATGGCATGC	For *OSH45* CRISPR construct
OSH45-U3-R	AAACGCATGCCATTACTGCTTCCA	
OSH45-U6a-F	GCCGAGAAAGCGAGCGATCCCTAA	
OSH45-U6a-R	AAACTTAGGGATCGCTCGCTTTCT	
OSH45-CX-F	ACGCACTACGTCTTGCTATT	For identifying *osh45* mutant
OSH45-CX-R	TCAAACCTGCTTTAGTTCATGC	
OSH45-GFP-inF	GAGCTCGGTACCCGGGGATCCAT GGGCGGCGGCGGCGAGGCGGA	For *35S::OSH45-GFP* construct
OSH45-GFP-inR	GCTCACCATGTCGACTCTAGACG AATTGTTATCACCTGCATTACT	
